# Long-Term Straw Return Combined with Tillage Management Systems Enhances Photosynthetic Capacity, Antioxidant Defense System, and Maize Yield

**DOI:** 10.3390/plants15203159

**Published:** 2026-10-15

**Authors:** Ju-Zhi Lv, Nan-Nan He, Peng-Ju Gao, Fa-Qiao Li, Xian-Jie Tan, Guo-Rong Tang, Rui-Ling Li, Xun-Bo Zhou

**Affiliations:** 1 Maize Research Institute of Guangxi Academy of Agricultural Sciences, Nanning 530007, China; 2 Guangxi Sub-Center of the National Maize Improvement Center, Nanning 530007, China; 3 Guangxi Key Laboratory of Agro-Environment and Agro-Products Safety, Key Laboratory of Crop Cultivation and Physiology, College of Agriculture, Guangxi University, Nanning 530004, China

**Keywords:** tillage systems, straw return, photosynthetic rate, antioxidant enzyme activities, maize production

## Abstract

Intensive maize production generates large quantities of crop residues, and the widespread practice of open-field straw burning poses serious environmental challenges. Sustainable agricultural intensification therefore requires effective residue management and tillage strategies that maintain crop productivity while minimizing environmental impacts. This study evaluated the effects of straw return (incorporation of chopped maize straw) versus straw removal, combined with four tillage practices: no-tillage (NT), rotary tillage (RT), sub-soiling (SS), and sub-soiling plus rotary tillage (SS + RT), on maize growth and yield in a subtropical double-cropping system in southern China. The results demonstrated that straw return significantly increased chlorophyll content and the net photosynthetic rate (Pn), while delaying the decline in leaf greenness and CO_2_ assimilation during the late growing season. Straw incorporation also enhanced the antioxidant defense system, increasing catalase (CAT) activity by approximately 34% and superoxide dismutase (SOD) activity by about 12% on average, while reducing oxidative damage compared with straw removal. Among the tillage treatments, SS-RT consistently produced the greatest improvements in photosynthetic performance and antioxidant enzyme activities, resulting in higher dry matter accumulation and grain yield. The combined application of straw return and SS-RT achieved the highest kernel number, 1000-kernel weight, and grain yield, increasing yield by up to 17% compared with no-tillage. These findings indicate that integrating straw return with a combined deep- and shallow-tillage system effectively enhances photosynthetic capacity, strengthens antioxidant defense, and improves maize productivity, providing a practical management strategy for intensive maize production.

## 1. Introduction

Maize (*Zea mays* L.) is one of the world’s most important cereal crops, with annual grain production exceeding 1.2 billion tons (FAOSTAT, http://www.fao.org/faostat/, accessed on 1 May 2026). China is the world’s second-largest maize producer, harvesting approximately 289 million tons of grain annually from more than 44 million ha, representing nearly 37% of the country’s total grain-growing area [1]. The Huang–Huai–Hai Plain (winter wheat–summer maize double-cropping system), Northeast Plain (spring maize), Southwestern Uplands (diverse maize rotations), and Northwest Plain are the country’s major maize-producing regions, collectively contributing approximately 216.03 Mt of maize annually [2,3]. These intensive production systems generate enormous quantities of crop residues. In traditional planting, to remove the maize straw, much of the straw has been disposed of through open-field burning, resulting in severe air pollution, greenhouse gas emissions, and degradation of agroecosystem health. To address these environmental concerns, the Chinese government prohibited open-field straw burning in 2014 and introduced policies promoting comprehensive straw utilization [4,5]. Consequently, in situ straw return has been widely adopted as an effective strategy to reduce environmental pollution, recycle nutrients, and improve soil quality. Developing residue management and tillage practices that simultaneously minimize environmental impacts and sustain high maize productivity has therefore become a major challenge for the sustainable intensification of maize-based production systems in China and worldwide.

Straw return, which involves retaining or incorporating chopped crop residues into the soil depending on the tillage system, provides substantial agronomic and ecological benefits [6,7]. Numerous studies have demonstrated that straw incorporation improves soil structure, increases soil organic matter, and enhances nutrient availability, thereby promoting crop growth and productivity [8,9,10]. Complete residue return has drawbacks, including temporary N immobilization, sowing and residue-management difficulties, phytotoxicity, disease risks, and possible increases in N_2_O emissions; however, these effects depend strongly on residue quality, C ratio, soil conditions, and management practices [11]. Appropriate straw return reduces soil water loss, increases soil water storage, enhances leaf photosynthesis, and promotes dry matter accumulation, ultimately leading to higher maize yields [11,12]. A global meta-analysis further reported that straw return increased soil available nitrogen, phosphorus, and potassium by approximately 14%, 10%, and 18%, respectively [13,14]. In addition to improving soil fertility and nutrient cycling, straw return substantially reduces the need for open-field burning, thereby mitigating environmental pollution. Consequently, straw incorporation has become a widely adopted practice for enhancing soil quality and sustaining maize productivity, particularly in wheat–maize double-cropping systems such as those of the Huang–Huai–Hai Plain. However, the benefits of straw return largely depend on the tillage system used to incorporate the residues into the soil [15,16].

Tillage practices play a fundamental role in regulating soil physical properties, water dynamics, nutrient availability, and crop performance. In China’s maize production systems, the most commonly adopted tillage methods include conventional rotary tillage, deep sub-soiling, sub-soiling combined with rotary tillage, and no-tillage [17,18,19]. These practices influence soil conditions through distinct mechanisms. Conventional rotary tillage effectively prepares the seedbed and incorporates surface residues but, when practiced continuously, may lead to the formation of a compact plough layer that restricts root growth and water movement [20]. In contrast, sub-soiling alleviates sub-soil compaction by disrupting dense soil layers, thereby improving soil aeration, water infiltration, root penetration, and access to deeper soil moisture [19,20,21,22]. Integrating deep sub-soiling with rotary tillage combines the advantages of both practices by simultaneously alleviating soil compaction and creating a well-prepared seedbed, providing favorable conditions for crop establishment and growth.

The physiological responses of maize to straw return and tillage are closely associated with changes in soil water availability, root development, and nutrient acquisition. Improvements in these factors can enhance stomatal conductance, chlorophyll content, and photosynthetic efficiency, thereby increasing carbon assimilation. Conversely, environmental stresses often induce excessive accumulation of reactive oxygen species (ROS), causing oxidative damage to cellular membranes and photosynthetic machinery. Antioxidant enzymes, including superoxide dismutase (SOD) and catalase (CAT), constitute the primary defense system against oxidative stress and therefore serve as important physiological indicators linking crop management practices with photosynthetic performance and grain yield. Several studies have shown that integrating straw return with appropriate tillage practices enhances these physiological processes. For example, ridge–furrow straw management significantly increased SOD and CAT activities while reducing malondialdehyde (MDA) accumulation and ROS levels, thereby protecting the photosynthetic apparatus in semi-arid maize systems [23]. Similarly, long-term straw return combined with an appropriate nitrogen application increased relative chlorophyll content (SPAD) values by approximately 2%, net photosynthetic rate (Pn) by about 8%, dry matter accumulation, and grain yield by nearly 15% compared with conventional management [24]. Sub-soiling has likewise been reported to improve photosynthetic rate and biomass accumulation in rain-fed maize on the Loess Plateau through enhanced soil water availability and root development [25]. Furthermore, Zhang et al. [26] demonstrated that four years of straw return increased soil urease, phosphatase, and invertase activities by up to 44.3%, thereby improving nutrient cycling and supporting higher photosynthetic capacity and maize productivity.

Despite these advances, important knowledge gaps remain regarding the interactive effects of straw management and tillage on maize physiology under contrasting agro-ecological conditions. Most previous studies have focused on temperate rain-fed or semi-arid environments, whereas comparable information from humid subtropical double-cropping maize systems remains limited. Differences in rainfall patterns and thermal regimes may substantially alter straw decomposition, nutrient mineralization, soil water dynamics, and root development, ultimately influencing leaf photosynthesis and antioxidant metabolism. Furthermore, the temporal responses of photosynthetic characteristics and antioxidant enzyme activities throughout different maize growth stages have rarely been investigated under factorial combinations of straw management and tillage practices.

Therefore, the present study evaluated the interactive effects of straw return and four tillage methods; no-tillage, rotary tillage, sub-soiling, and sub-soiling combined with rotary tillage on maize photosynthetic characteristics, antioxidant enzyme activities, dry matter accumulation, and grain yield in a representative subtropical double-cropping production system. We hypothesized that integrating straw return with sub-soiling plus rotary tillage would improve photosynthetic performance, strengthen antioxidant defense mechanisms, and ultimately increase maize productivity compared with conventional residue management and tillage practices. The findings will provide a physiological basis for optimizing residue and tillage management strategies that enhance maize productivity while reducing environmental impacts, thereby contributing to the sustainable intensification of maize-based cropping systems.

## 2. Materials and Methods

### 2.1. Site Description

**Figure 1:**
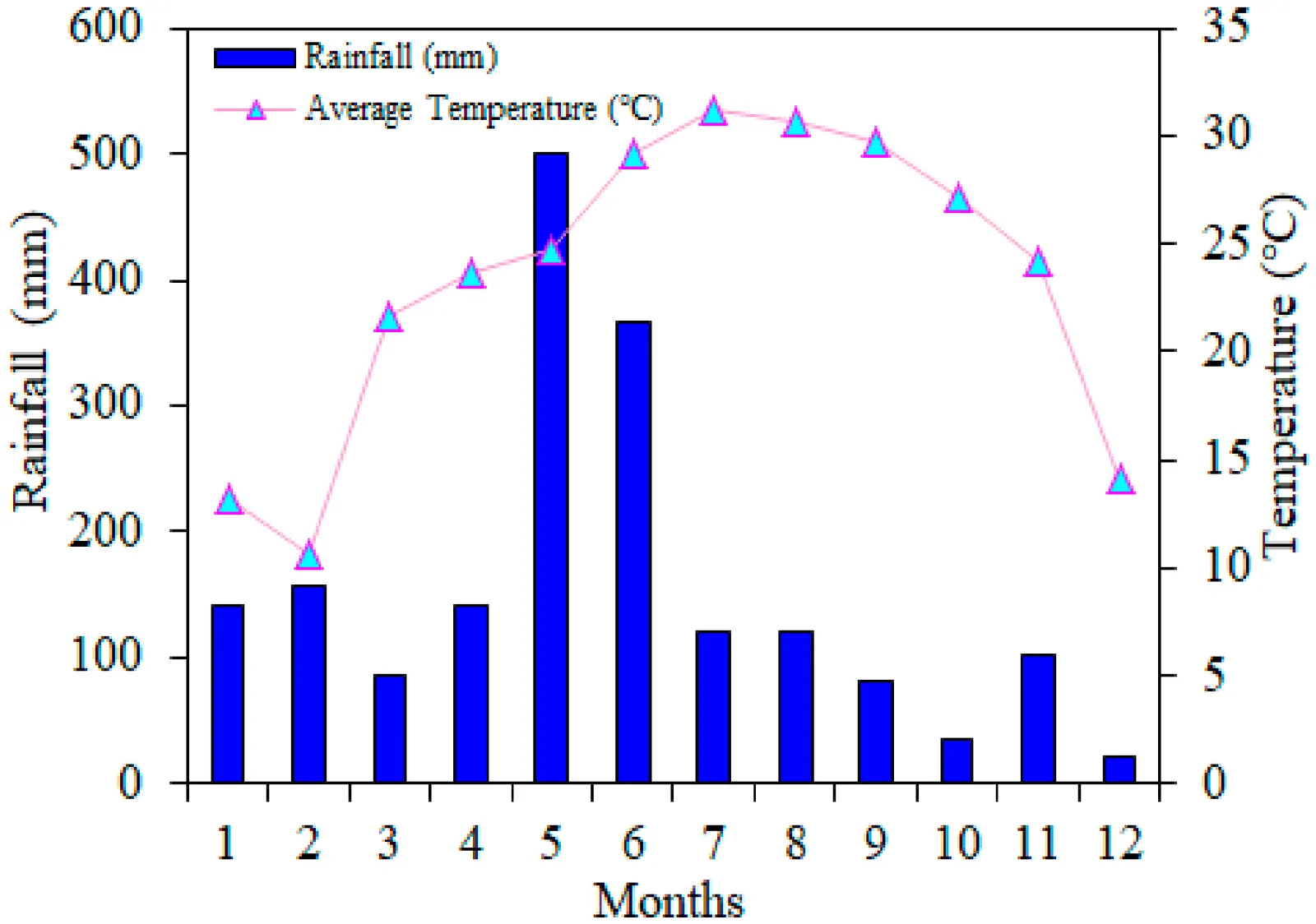
Monthly cumulative rainfall and temperature in 2023.

A long-term field experiment was conducted at the Maize Research Institute, Guangxi Academy of Agricultural Sciences in Nanning, China (22°36′34″ N, 108°14′33″ E). The region is characterized by a humid subtropical monsoon climate with abundant thermal resources and concurrent rainfall and heat, facilitating a double-cropping maize system (spring and autumn maize). The annual mean temperature is approximately 23.8 °C, with a mean annual precipitation of about 1245 mm (World Weather Information Service of the World Meteorological Organization; WMO) (Figure 1). The soil is clay in texture, exhibiting high water and fertilizer retention capacity, but is prone to compaction under improper tillage. Prior to the experiment, the tested soil properties (0–20 cm depth) showed as soil pH of 6.46, soil organic carbon (SOC) of 12.5 g kg^−1^ (equivalent to 21.46 g kg^−1^ soil organic matter using a conversion factor of 1.724), and total nitrogen (TN), total phosphorus (TP), and total potassium (TK) of 0.85, 0.15, and 23.27 g kg^−1^, respectively. Soil classification was based on international systems, ideally the World Reference Base (WRB) or the USDA Soil Taxonomy—specifically, clay soil.

### 2.2. Experimental Design and Field Management

The experiment was laid out in a split-plot arrangement. Straw management was assigned to main plots with two treatments: straw return (SR), where aboveground maize residues from the previous season were chopped and returned to the field; and traditional planting (TP), where straw residues were removed. Tillage practice was assigned to sub-plots with four levels: no-tillage (NT, 0 cm), rotary tillage (RT, 25 cm), sub-soiling (SS, 35 cm), and sub-soiling followed by rotary tillage (SS-RT, 35 cm + 25 cm). Under RT/SS/SS-RT, returned straw was incorporated into the soil, whereas under NT, it was retained on the soil surface. Rotary tillage is performed annually before maize planting, while sub-soiling is performed every other year before maize planting. Each treatment combination was replicated three times (8 treatments × 3 replicates = 24 plots). Each plot measured 20.8 m × 11.2 m (233 m^2^).

The maize hybrid Guidan 162 was used as a test crop. The experiment was established in 2018 followed a double-cropping system; data reported here are from the sixth year (2023), with spring maize sown on 20 March and autumn maize on 25 August. The experiment, which began in 2018, has consistently grown maize, with no prior rotation; each treatment returned all above-ground parts of the previous season’s maize harvested to the field. Maize was planted at 65 cm row spacing and 28 cm within-row spacing. Seedlings were thinned at the 3–4 leaf stage to a final density of 52,500 plants ha^−1^. A single basal application of stabilized compound fertilizer (Entek; N: P_2_O_5_: K_2_O = 21:7:11) was applied at 900 kg ha^−1^ to all plots, and no additional fertilizer was applied during the growing season. Pest, disease, and weed management followed local commercial practices, and guard rows were established around the experimental area. After harvesting, the entire amount of straw was returned to the field on a fresh-weight basis during both spring and autumn, and chopped into approximately 5-cm lengths. Irrigation was carried out 3 times in 2023.

### 2.3. Photosynthetic Traits, Biomass Sampling, and Leaf Collection

The leaf area index (LAI) was measured at the three-leaf growth stage (V3), six-leaf stage (V6), twelve-leaf stage (V12), silking stage (R1), and physiological maturity (R6) stage. For each treatment, 12 plants with uniform growth were selected. Individual leaf area was estimated using the length–width coefficient method (leaf area = length × width × 0.75), and the LAI was calculated based on leaf area per plant and plant density. The relative chlorophyll content (SPAD) was measured at the same stages using a SPAD-502 chlorophyll meter (Konica Minolta, Tokyo, Japan) on the ear leaf.

Gas exchange parameters were measured between 9:30 and 11:30 using a LI-6800 portable photosynthesis system (LI-COR, Lincoln, NE, USA) after instrument stabilization. Chamber settings were as follows: photosynthetic photon flux density 1500 μmol m^−2^ s^−1^, relative humidity 70%, leaf temperature 30 °C, flow rate 500 μmol s^−1^, and CO_2_ concentration 400 μmol mol^−1^. For each treatment, ear leaves from 12 randomly selected plants were measured. Antioxidant-related assays were conducted at the V3 and V6 developmental stages, while ear leaves were collected at the V12, R1, and R6 growth stages between 9:00 and 11:00 on clear days, immediately frozen in liquid nitrogen, and stored at −80 °C until analysis.

At R1 and R6, nine plants per treatment were sampled and separated into stem, leaf, and ear. Samples were oven-killed at 105 °C for 1 h and dried at 80 °C to constant weight for dry matter determination [27,28].

### 2.4. Physiological and Biochemical Assays

Fully expanded leaves were collected at the V3 and V6 developmental stages, while ear leaves were collected at the V12, R1, and R6 growth stages, to determine the physiological and biochemical assays. Frozen leaf samples were weighed (0.1000 g) into 2 mL tubes with two 3 mm stainless-steel beads and homogenized using a high-throughput tissue grinder (Xinyi-48N, Ningbo Scientz, Ningbo, China). After adding 1 mL extraction buffer, homogenates were centrifuged at 8000× *g* for 10 min at 4 °C (Fresco 21, Thermo Scientific, Waltham, MA, USA). The supernatant was used for assays. Superoxide dismutase (SOD), peroxidase (POD), catalase (CAT), malondialdehyde (MDA), and proline were determined using commercial assay kits (Solarbio, Beijing, China; kit codes BC0175, BC0095, BC0205, BC0025, and BC0295, respectively) following the manufacturer’s instructions.

### 2.5. Yield Determination

At maturity, plants were harvested from a 20 m^2^ central area in each plot to measure grain yield at 14% moisture content. For yield component analysis, 20 representative plants per plot were sampled to measure plant height, stem diameter, ear height, ear length, ear diameter, barren tip length, number of rows per ear, kernels per row, kernel number per ear, and thousand-grain weight.

### 2.6. Statistical Analysis

Data were processed using Microsoft Excel 2019 and analyzed with IBM SPSS Statistics 21.0. An randomize complete block design (RCBD) design was used to test the effects of straw and tillage management. The three-replicate data for each treatment were analyzed, and significant differences between treatments were determined based on the least significant difference (LSD) test. For all the parameters, the analysis of variance (ANOVA) (straw management × tillage) was conducted using IBM SPSS Statistics 21.0 (SPSS Inc., Chicago, IL, USA), with the split-plot error structure, replication/block, the main-plot error, and the sub-plot error appropriately incorporated into the statistical model. Differences were considered significant when *p* < 0.05. The means among treatments were compared based on the least significant difference (LSD) test. All the analyses were performed with a confidence level of 95% by using SPSS 13.0 (SPSS Inc. Chicago, IL, USA), and data obtained from each sampling event (growth stage) were analyzed separately. Data analyses were conducted using Excel 2019 and SPSS 13.0, and all figures were prepared using Origin 2019 and Sigma Plot 12.5. Regression correlation analysis of yield with photosynthetic physiological characteristics was conducted using SPSS 13.0 (SPSS Inc. Chicago, IL, USA).

## 3. Results

### 3.1. Leaf Area Index and Chlorophyll Contents

**Figure 2:**
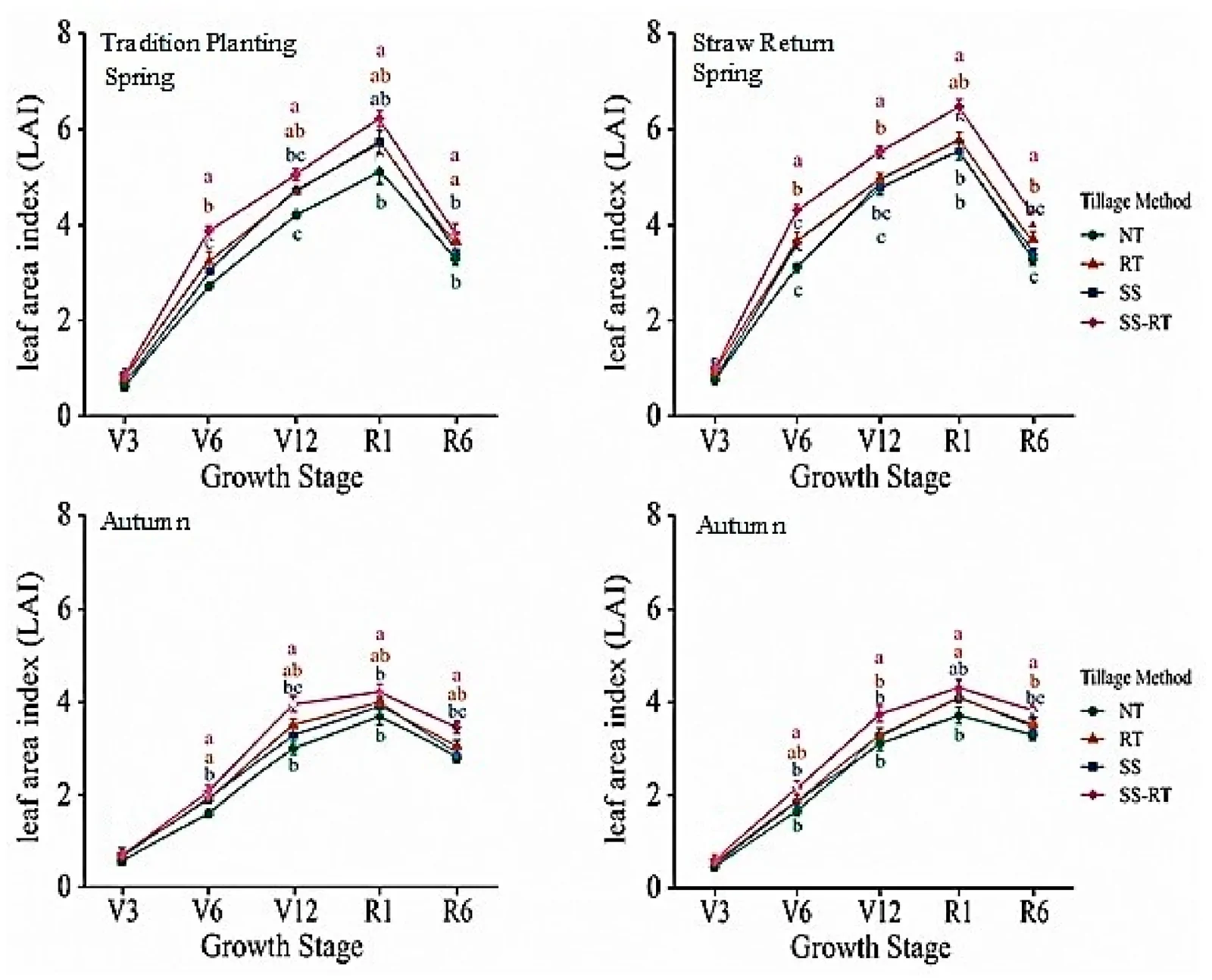
Effect of straw treatment combined with tillage methods on the leaf area index (LAI) of maize. The bars are the standard error (SE). Tillage practice was assigned to sub-plots with four levels: NT: no-tillage (0 cm), RT: rotary tillage (25 cm), SS: sub-soiling (35 cm), and SS-RT: sub-soiling followed by rotary tillage (35 cm + 25 cm). Different lower-case letters for the same growth stage between different tillage methods indicate significant differences at *p* < 0.05 (*n* = 3).

For spring maize, the mean leaf area index (LAI) under straw return (SR) and traditional planting to remove the straw (TP) was 3.81 and 3.55, respectively (Figure 2). Under SR, the combined sub-soiling plus rotary tillage (SS-RT), rotary tillage (RT), sub-soiling (SS), and no-tillage (NT) treatments produced average LAI values of 4.29, 3.78, 3.66, and 3.51, whereas the corresponding values under TP were 3.88, 3.63, 3.46, and 3.24, respectively. For both management systems, SS-RT yielded a significantly higher LAI than NT (*p* < 0.05), representing increases of 18.16% and 16.54%. Within any given phonological stage, SR consistently delivered a greater LAI than TP under the same tillage regime. Across the entire V3–R6 growth period, the LAI ranking was SS-RT > RT > SS > NT, and tillage treatment significantly affected the LAI at every developmental stage (*p* < 0.05). In the autumn season, the mean LAI under SR was 3.02, whereas TP recorded an average LAI of 2.74, an increase of 10.22% attributable to SR (*p* < 0.05). Between the R1 and R6 growth stages, the LAI declined by 4.43%, 8.72%, 11.72%, and 8.25% for SR-based SS-RT, RT, SS, and NT treatments, respectively, compared with reductions of 13.74%, 22.09%, 23.45%, and 23.66% under the corresponding TP systems. Thus, SR markedly attenuated the rate of LAI decline from R1 to R6. During late development, both straw management and tillage exerted significant effects on the LAI (*p* < 0.05), collectively helping to sustain a more gradual decrease in canopy leaf area. Across two consecutive seasons, the same pattern was confirmed: the LAI under SR remained significantly higher than under TP throughout all growth stages (*p* < 0.05), and the ranking among tillage treatments consistently followed SS-RT > RT > SS > NT.

**Figure 3:**
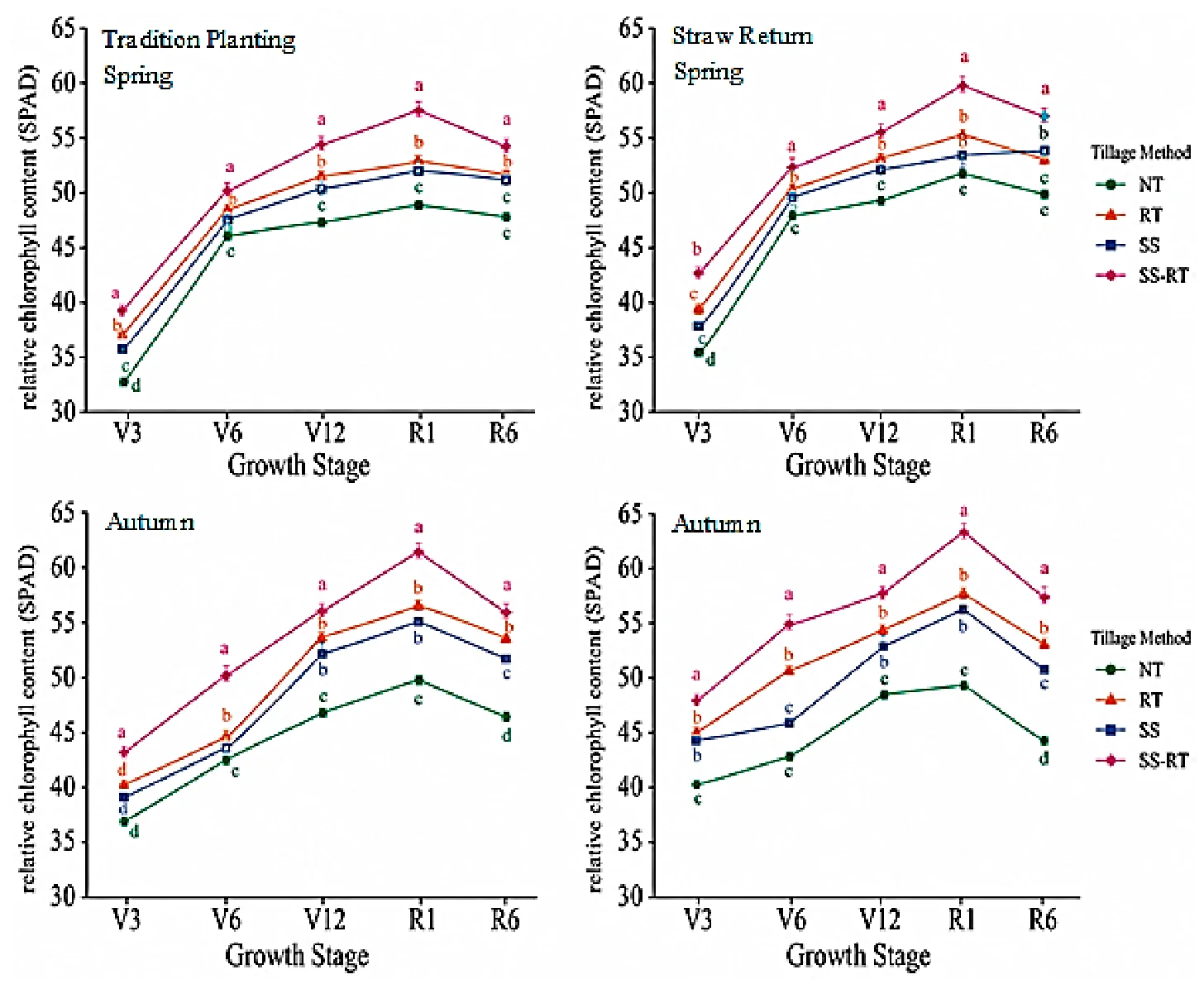
Effect of straw treatment combined with tillage methods on the relative chlorophyll content (SPAD) of maize. The bars are the standard error (SE). Tillage practice was assigned to sub-plots with four levels: NT: no-tillage (0 cm), RT: rotary tillage (25 cm), SS: sub-soiling (35 cm), and SS-RT: sub-soiling followed by rotary tillage (35 cm + 25 cm). Different lower-case letters for the same growth stage between different tillage methods indicate significant differences at *p* < 0.05 (*n* = 3).

The relative chlorophyll content (SPAD) rises during early development in both spring and autumn, reaches a maximum at the R1 stage, and declines toward maturity (Figure 3). In spring, the mean SPAD values were 50.51 and 47.98 under SR and TP, respectively. Under TP, the combined sub-soiling plus rotary tillage treatment (SS-RT) produced 2.39%, 3.97%, and 10.30% SPAD values, higher than those under rotary tillage (RT), sub-soiling (SS), and no-tillage (NT); under SR, the corresponding advantages were 3.96%, 4.11%, and 11.43%. At R1, SPAD averaged 59.50 under SR and 57.40 under TP; by R6, these values declined to 56.70 and 53.53. For SS-RT, the R1-to-R6 decrease amounted to 4.71% under SR and 6.74% under TP, showing that straw return slowed late-season chlorophyll loss. In autumn, mean SPAD values were 52.06 under SR and 49.17 under TP. Within SR, SS-RT, RT, SS, and NT yielded SPAD values of 56.15, 52.51, 51.10, and 48.49, so SS-RT surpassed RT, SS, and NT by 6.92%, 9.88%, and 15.78%. Across two consecutive seasons, SPAD increased early and declined later; SR consistently produced significantly higher values than TP at every stage (*p* < 0.05). Straw management and tillage each significantly affected SPAD (*p* < 0.05), and their combination helped maintain higher chlorophyll content during late reproductive development, indicating that straw return coupled with appropriate tillage helps sustain canopy greenness and photosynthetic capacity in maize.

### 3.2. Intercellular CO_2_ Concentration

**Figure 4:**
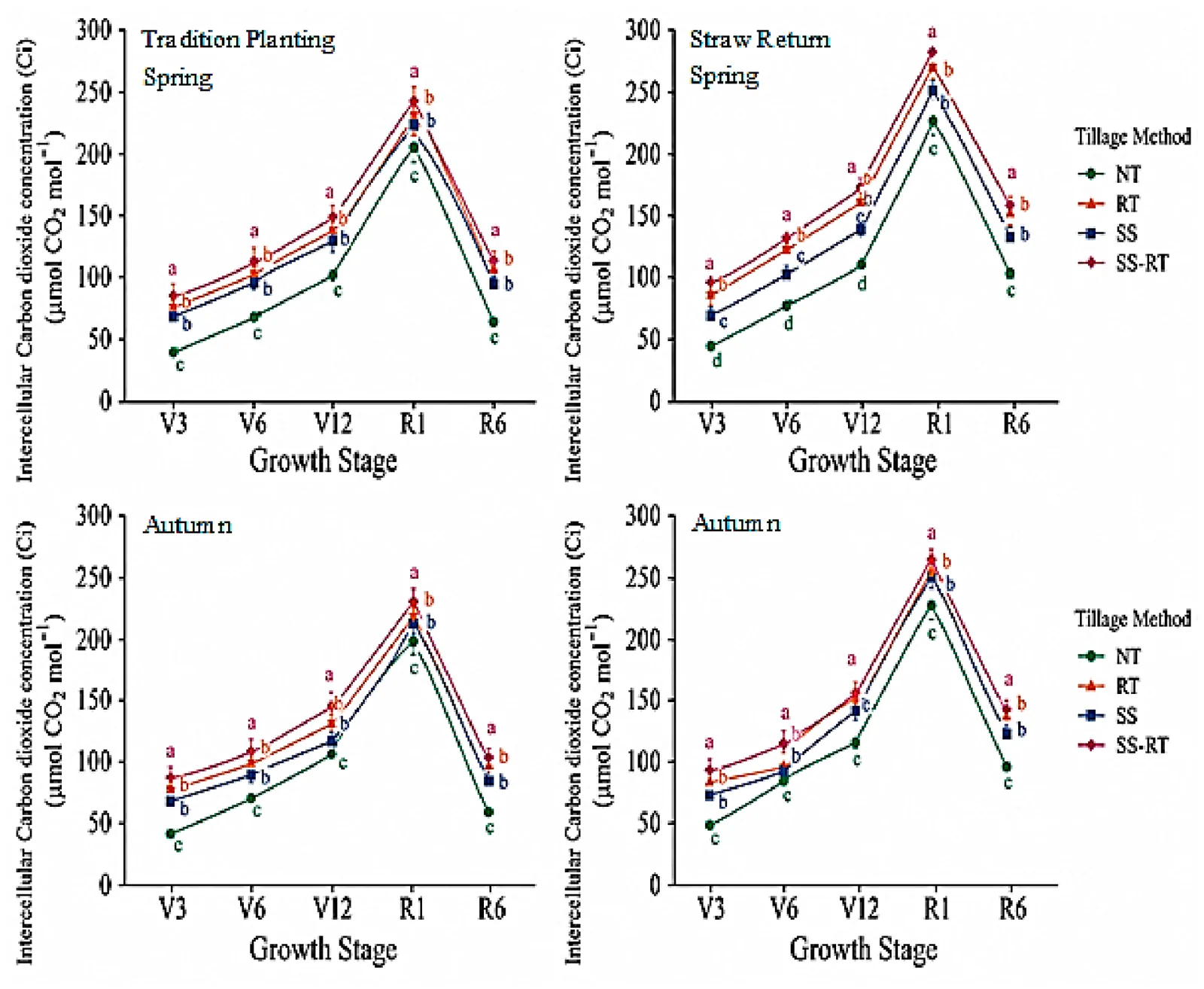
Effect of straw treatment combined with tillage methods on the intercellular carbon dioxide concentration (Ci) of maize. The bars are the standard error (SE). Tillage practice was assigned to sub-plots with four levels: NT: no-tillage (0 cm), RT: rotary tillage (25 cm), SS: sub-soiling (35 cm), and SS-RT: sub-soiling followed by rotary tillage (35 cm + 25 cm). Different lower-case letters for the same growth stage between different tillage methods indicate significant differences at *p* < 0.05 (*n* = 3).

The intercellular CO_2_ concentration (Ci) of spring maize was 16.70% higher under SR than under TP (Figure 4). Relative to TP, SR increased Ci by 19.36%, 22.55%, 19.29%, and 19.94% for SS-RT, RT, SS, and NT, respectively. At the R1 stage, TP plots showed that SS-RT raised Ci by 2.90%, 7.73%, and 8.21% compared with RT, SS, and NT, while under SR, the corresponding gains were 0.40%, 4.69%, and 16.67%. Across all growth stages, Ci was consistently higher under SR than under TP, and within the SR regime, the SS-RT treatment produced significantly greater Ci increases than the other tillage practices (*p* < 0.05). During the autumn growing season, the mean Ci was 139.90 µmol CO_2_ mol^−1^ under SR, which was 17.68% higher than the 118.88 µmol CO_2_ mol^−1^ recorded under TP. At R6, the corresponding figures were 140.29, 128.57, 127.76, and 99.43 µmol CO_2_ mol^−1^ (SR) versus 102.74, 95.77, 95.12, and 63.72 µmol CO_2_ mol^−1^ (TP). In both management systems, the SS-RT treatment produced significantly higher Ci than RT, SS, or NT. Results from the two-season experiment showed that straw return (145.48 µmol CO_2_ mol^−1^) increased the Ci by 18.90% compared with traditional planting to remove the straw (122.36 µmol CO_2_ mol^−1^) (Table 1). Across all tillage treatments, spring maize exhibited a Ci 7.00% higher than that of autumn maize (*p* < 0.05), indicating that straw management, the tillage system, and the season are each important determinants of Ci. Because CO_2_ is a key substrate for photosynthesis, increasing its intercellular concentration within an appropriate range is conducive to enhanced carbon assimilation and, consequently, greater dry matter accumulation.

### 3.3. Net Photosynthetic Rate

**Figure 5:**
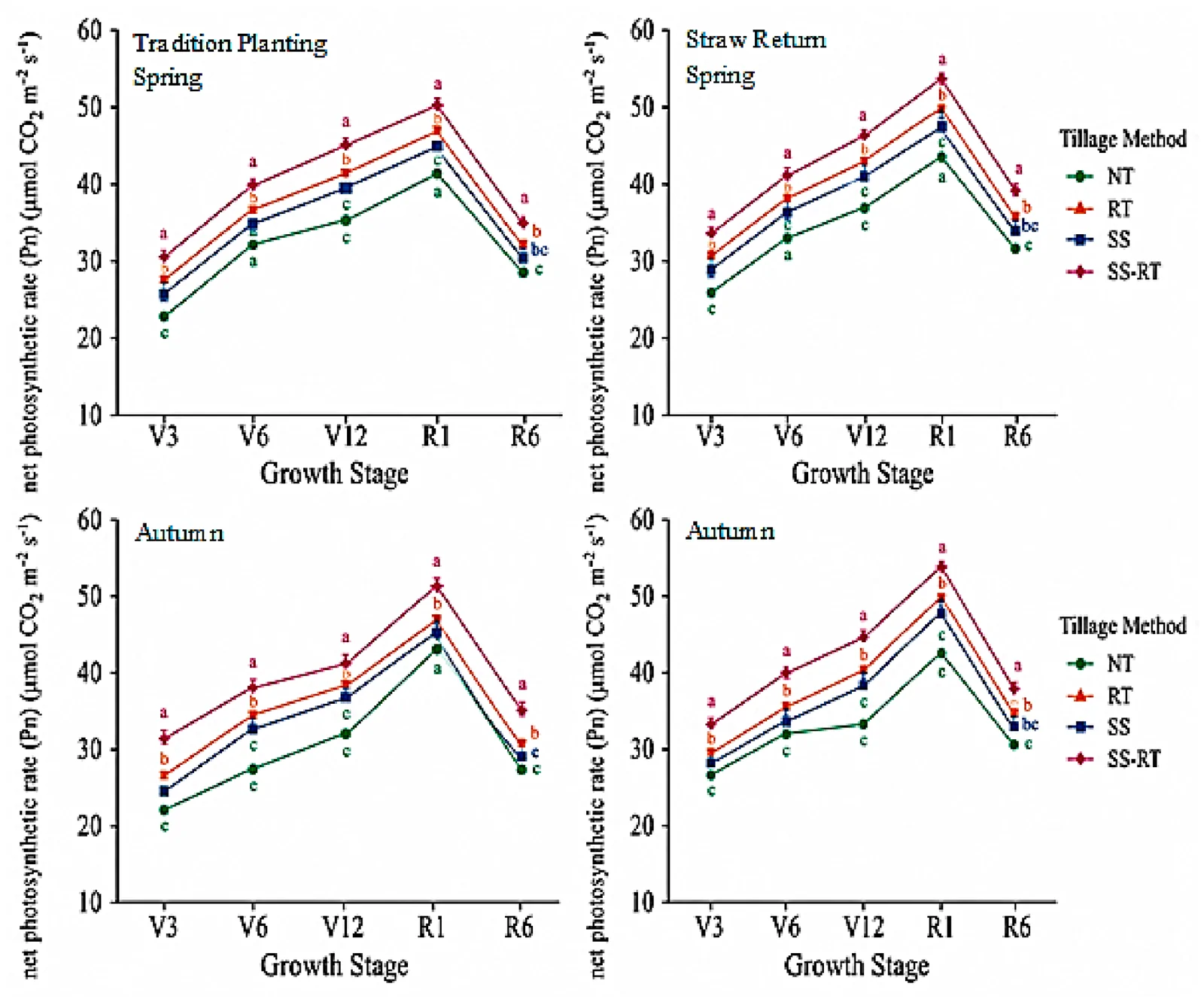
Effect of straw treatment combined with tillage methods on the net photosynthetic rate (Pn) of maize. The bars are the standard error (SE). Tillage practice was assigned to sub-plots with four levels: NT: no-tillage (0 cm), RT: rotary tillage (25 cm), SS: sub-soiling (35 cm), and SS-RT: sub-soiling followed by rotary tillage (35 cm + 25 cm). Different lower-case letters for the same growth stage between different tillage methods indicate significant differences at *p* < 0.05 (*n* = 3).

During the spring maize growing season, SR increased Pn by 7.51% relative to TP (Figure 5). The Pn values at the V3, V6, V12, R1, and R6 stages were 28.78, 37.63, 41.88, 50.13, and 36.46 µmol CO_2_ m^−2^ s^−1^ under SR and 26.09, 35.24, 40.49, 46.49, and 32.49 µmol CO_2_ m^−2^ s^−1^ under TP, corresponding to increases of 10.30%, 6.77%, 3.42%, 7.82%, and 12.21%, respectively. At R6, SR raised Pn by 10.82% (SS-RT), 13.06% (RT), 11.83% (SS), and 13.34% (NT) compared with TP. These results indicate that combining straw return with appropriate tillage helps maintain higher Pn, thereby favoring the synthesis and accumulation of post-anthesis assimilates. In autumn maize, the Pn first increased and then declined, peaking at the R1 stage. During the pre-anthesis period (V3–V12), SS-RT under SR recorded Pn values 10.13%, 11.64%, and 18.89% higher than those of RT, SS, and NT, respectively; under TP, the corresponding advantages were 8.71%, 11.78%, and 25.82%. During the post-anthesis period (R1–R6), SS-RT under SR exceeded RT, SS, and NT by 5.04%, 7.69%, and 16.63%, whereas under TP, the increases were 9.78%, 13.76%, and 15.12%. As indicated by the two-season data in Table 2, SR, tillage practice, and their interaction all exerted significant effects on Pn. The combination of SR with SS-RT effectively sustained a higher post-anthesis Pn, thereby enhancing photosynthesis and promoting greater dry matter accumulation.

### 3.4. Stomatal Conductance

**Figure 6:**
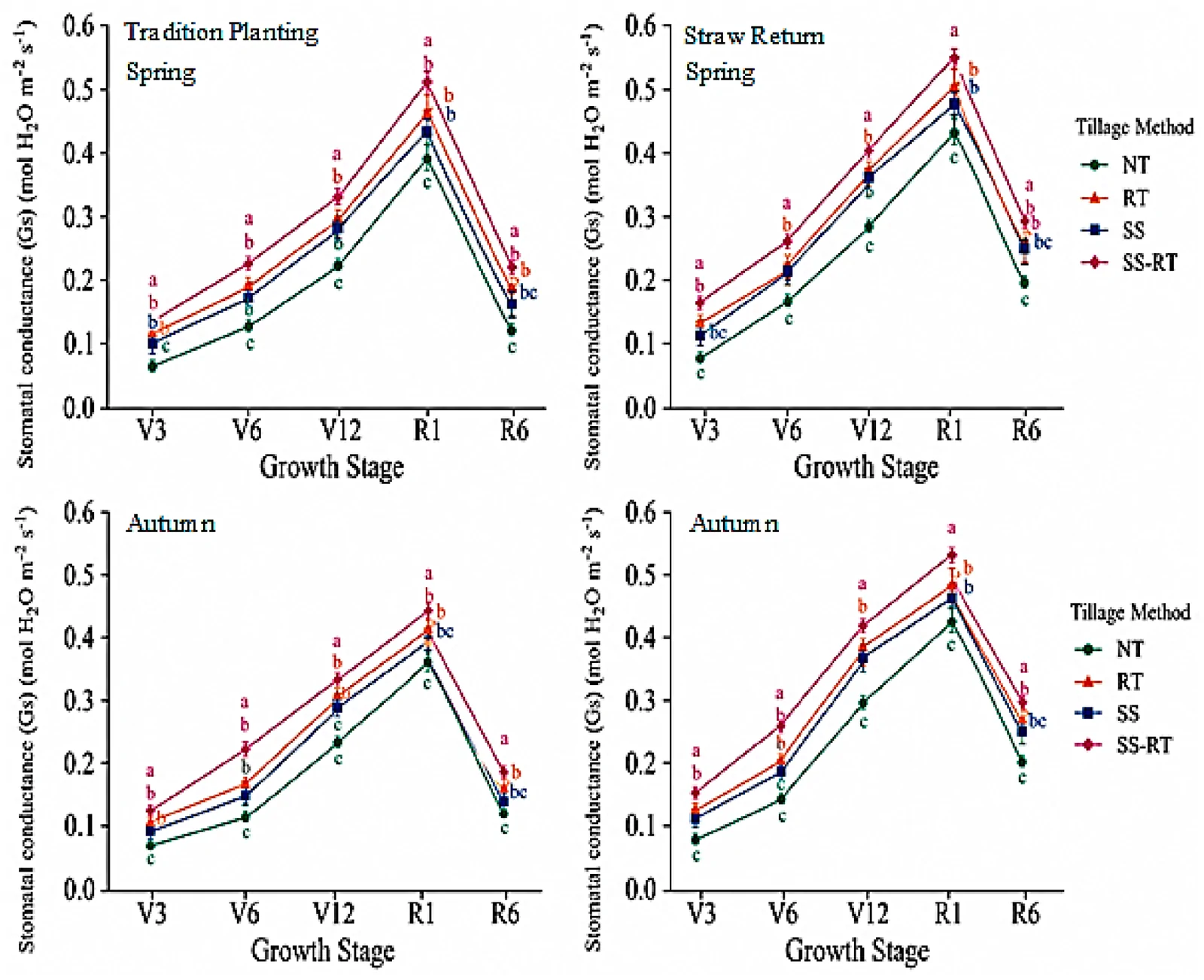
Effect of straw treatment combined with tillage methods on the stomatal conductance (Gs) of maize. The bars are the standard error (SE). Tillage practice was assigned to sub-plots with four levels: NT: no-tillage (0 cm), RT: rotary tillage (25 cm), SS: sub-soiling (35 cm), and SS-RT: sub-soiling followed by rotary tillage (35 cm + 25 cm). Different lower-case letters for the same growth stage between different tillage methods indicate significant differences at *p* < 0.05 (*n* = 3).

Stomatal conductance (Gs) exhibited a clear inverted-“V” pattern over the spring season, reaching its maximum at the R1 stage (Figure 6). At this stage, SR resulted in a mean Gs of 0.31 mol H_2_O m^−2^ s^−1^, which was 29.17% higher than that under traditional planting to remove the straw (TP; 0.24 mol H_2_O m^−2^ s^−1^; *p* < 0.05). Under TP, all tillage treatments differed significantly (*p* < 0.05); relative to NT, Gs increased by 12.44%, 4.06%, and 2.08% under SS-RT, RT, and SS, respectively. At R6, Gs under SS-RT, RT, SS, and NT averaged 0.21, 0.19, 0.17, and 0.16 mol H_2_O m^−2^ s^−1^ under TP and 0.32, 0.28, 0.28, and 0.21 mol H_2_O m^−2^ s^−1^ under SR. Across all tillage treatment, SR maintained significantly higher post-anthesis Gs than TP (*p* < 0.05). During the autumn season, Gs also followed an inverted-“V” trajectory, with seasonal means of 0.30 mol H_2_O m^−2^ s^−1^ under SR and 0.23 mol H_2_O m^−2^ s^−1^ under TP. At R1, SR enhanced Gs by 12.44%, 12.36%, 13.96%, and 8.78% relative to TP for SS-RT, RT, SS, and NT; the corresponding gains at R6 were 16.97%, 23.56%, 23.89%, and 17.34%. Across all tillage systems, SR consistently produced significantly higher Gs than TP (*p* < 0.05), indicating that straw return, particularly when combined with SS-RT, is conducive to maintaining stomatal conductance before and after anthesis. Results from the two-season maize experiment showed that SR increased mean Gs by 27.24% relative to TP (*p* < 0.05) (Table 3). Among tillage treatments, Gs under combined SS-RT exceeded that under RT, SS, and NT by 9.98%, 14.87%, and 27.44%, respectively (*p* < 0.05). The degree of stomatal opening was also modulated by seasonal climatic conditions.

### 3.5. Transpiration Rate

**Figure 7:**
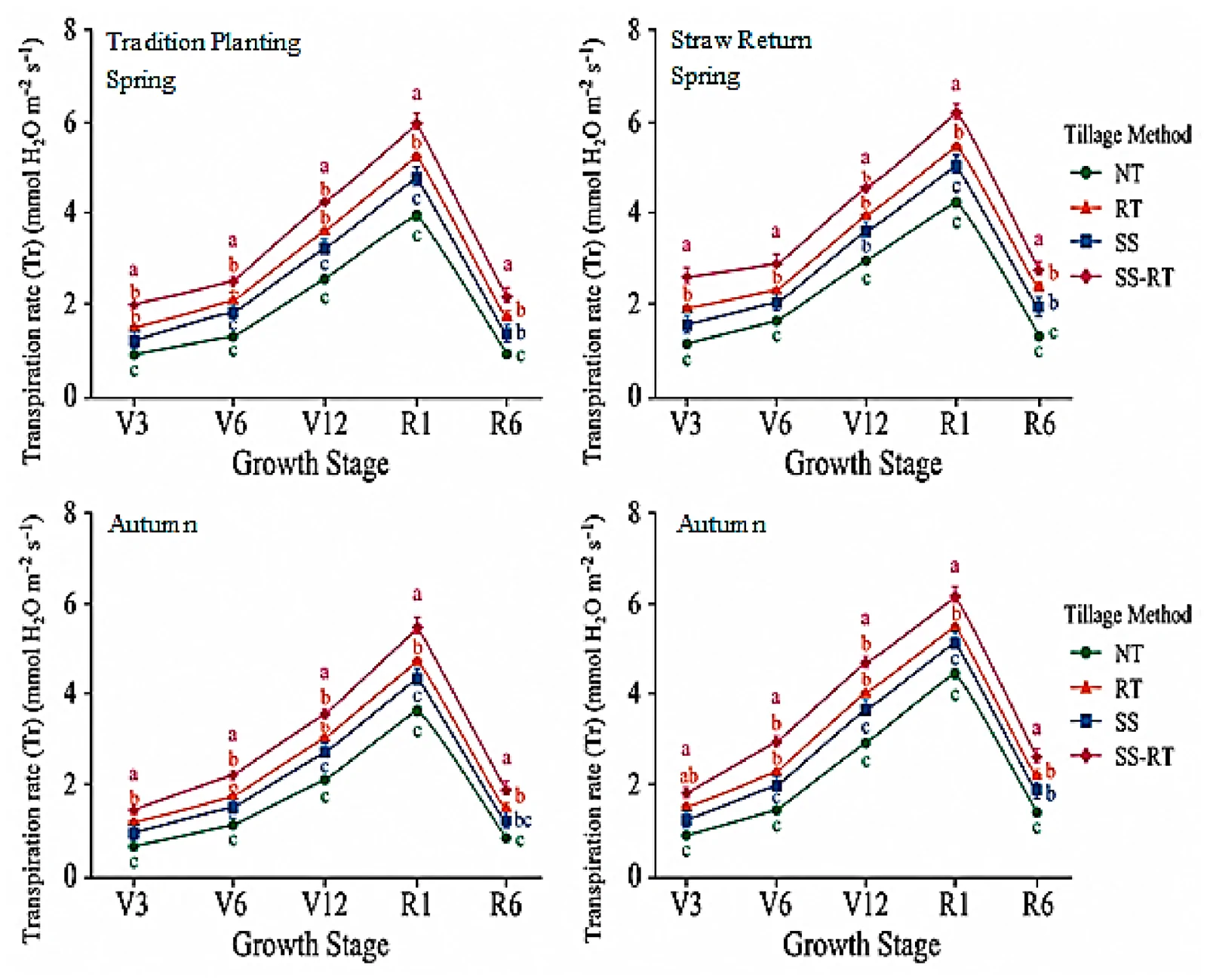
Effect of straw treatment combined with tillage methods on the transpiration rate (Tr) of maize. The bars are the standard error (SE). Tillage practice was assigned to sub-plots with four levels: NT: no-tillage (0 cm), RT: rotary tillage (25 cm), SS: sub-soiling (35 cm), and SS-RT: sub-soiling followed by rotary tillage (35 cm + 25 cm). Different lower-case letters for the same growth stage between different tillage methods indicate significant differences at *p* < 0.05 (*n* = 3).

During the spring growing season, the transpiration rate (Tr) increased and then declined, showed an inverted-“V” pattern with a peak at the R1 stage (Figure 7). In both straw treatments, the tillage sequence ranking was SS-RT > RT > SS > NT, with all differences significant (*p* < 0.05). Across tillage system, SR consistently produced higher Tr than TP (*p* < 0.05). During the autumn season, Tr averaged 2.96 mmol H_2_O m^−2^ s^−1^ under SR and 2.42 mmol H_2_O m^−2^ s^−1^ under TP, representing a 22.45% increase with SR (*p* < 0.05). Mean Tr at V3, V6, V12, R1, and R6 was 1.18, 1.69, 2.93, 4.80, and 1.55 mmol H_2_O m^−2^ s^−1^ under TP and 1.54, 2.13, 3.64, 5.54, and 1.95 mmol H_2_O m^−2^ s^−1^ under SR. Tr again rose and then fell over time, peaking at R1. Before anthesis (V3–V12), SR (2.44 mmol H_2_O m^−2^ s^−1^) raised Tr by 26.20% compared with TP (1.93 mmol H_2_O m^−2^ s^−1^); after anthesis (R1–R6), SR (3.75 mmol H_2_O m^−2^ s^−1^) surpassed TP (3.17 mmol H_2_O m^−2^ s^−1^) by 17.97%. SS-RT consistently produced significantly higher Tr than RT, SS, and NT (*p* < 0.05), confirming that the combination of straw return and appropriate tillage, especially SS-RT, enhances transpiration both before and after flowering (Figure 7). Across the two seasons, SR increased the mean maize Tr by 18.75% in spring, 22.45% in autumn, and 20.50% on an annual basis compared with TP (Table 4). Across the year, the Tr under combined SS-RT exceeded that under RT, SS, and NT by 11.78%, 17.13%, and 25.61%, respectively, yielding the overall hierarchy SR > TP and SS-RT > RT > SS > NT. Spring maize exhibited significantly higher Tr (2.93 mmol H_2_O m^−2^s^−1^) than autumn maize (2.69 mmol H_2_O m^−2^ s^−1^), an increase of 8.99% (*p* < 0.05), confirming that straw management, tillage practice, and season each have significant effects on Tr.

### 3.6. Dry Matter Accumulation

**Figure 8:**
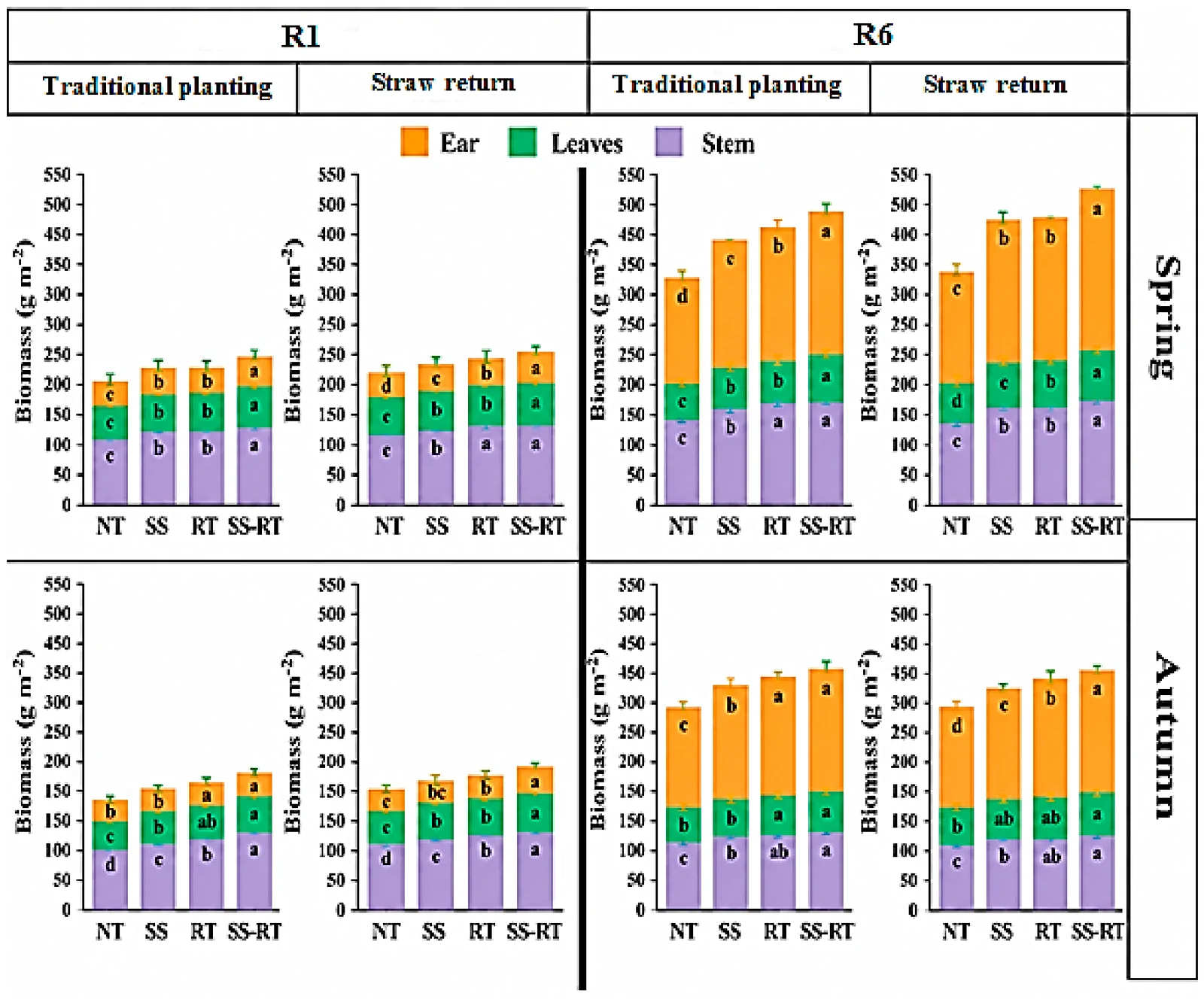
Effects of straw treatment combined with tillage methods on the dry matter accumulation of maize. The bars are the standard error (SE). Tillage practice was assigned to sub-plots with four levels: NT: no-tillage (0 cm), RT: rotary tillage (25 cm), SS: sub-soiling (35 cm), and SS-RT: sub-soiling followed by rotary tillage (35 cm + 25 cm). Different lower-case letters for the same growth stage between different tillage methods indicate significant differences at *p* < 0.05 (*n* = 3).

At the R1 stage in spring, SR increased stem dry matter accumulation in the SS-RT, RT, SS, and NT treatments by 3.84%, 7.21%, 4.19%, and 10.33%, respectively, compared with TP; the corresponding increases for leaves were 4.18%, 4.51%, 5.22%, and 8.72%, and for ears, 11.21%, 14.44%, 9.40%, and 10.00%. By R6, SR raised stem dry matter by 8.78%, 3.15%, 8.17%, and 3.71%; leaf dry matter by 6.17%, 11.51%, 6.67%, and 10.81%; and ear dry matter by 8.92%, 3.83%, 7.68%, and 8.23% in the same tillage sequence. In autumn, SR increased stem dry matter accumulation at R6 by 5.88%, 5.59%, 5.90%, and 6.02% for SS-RT, RT, SS, and NT, respectively; leaf dry matter rose by 3.78%, 4.20%, 9.41%, and 14.24%, and ear dry matter by 5.77%, 4.14%, 2.84%, and 6.12%. Under SR, ear dry matter in SS-RT was significantly higher than in RT, SS, and NT (*p* < 0.05), exceeding them by 5.02%, 10.00%, and 25.58%, respectively. Across the two seasons (Figure 8), SR consistently enhanced dry matter accumulation in all organs. At R1, the order of accumulation was stem > leaf > ear, whereas at R6, it shifted to ear > stem > leaf, indicating a progressive transfer of dry matter from stems and leaves to the ears.

### 3.7. Antioxidant Enzyme Activities in Maize

**Figure 9:**
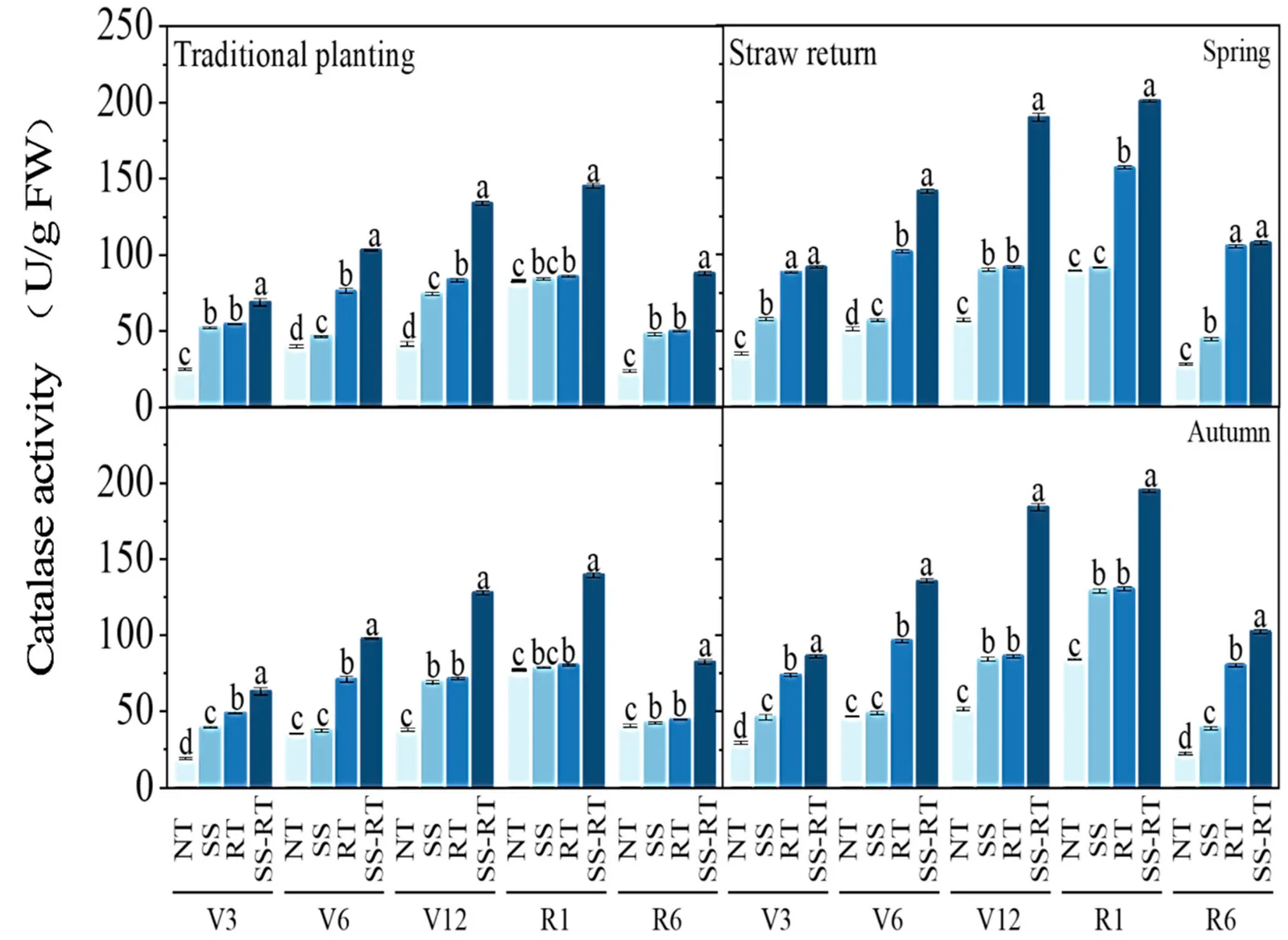
Effects of straw treatment combined with tillage methods on the catalase activity of maize. The bars are the standard error (SE). Tillage practice was assigned to sub-plots with four levels: NT: no-tillage (0 cm), RT: rotary tillage (25 cm), SS: sub-soiling (35 cm), and SS-RT: sub-soiling followed by rotary tillage (35 cm + 25 cm). Different lower-case letters for the same growth stage between different tillage methods indicate significant differences at *p* < 0.05 (*n* = 3).

During the spring growth period, catalase (CAT) activity averaged 88.55 U g^−1^ under SR, 35.73% higher than the 65.24 U g^−1^ recorded under TP. Enzyme activity rose and then declined over the season, peaking at R1. At R1, mean CAT activity was 134.75 U g^−1^ under SR and 93.92 U g^−1^ under TP; at R6, SR exceeded TP by 20.16% (*p* < 0.05). Within SR, CAT activity in SS-RT and RT was significantly higher than in SS and NT (*p* < 0.05), whereas SS-RT and RT did not differ (*p* > 0.05). For autumn maize, CAT activity averaged 93.85 U g^−1^ under SR and 70.45 U g^−1^ under TP, an increase of 33.21% with SR. Activity again rose to a maximum at R1 and then declined, with SS-RT consistently surpassing RT, SS, and NT. Relative to TP, SR increased CAT activity by 26.31%, 35.58%, 10.06%, and 18.22% in SS-RT, RT, SS, and NT, respectively (Figure 9). Across seasons, the interaction between straw management and tillage significantly affected leaf CAT activity (*p* < 0.05). On average, straw return raised CAT activity by 34.43% relative to traditional planting to remove the straw, confirming that straw return markedly enhances enzymatic protection (*p* < 0.05).

**Figure 10:**
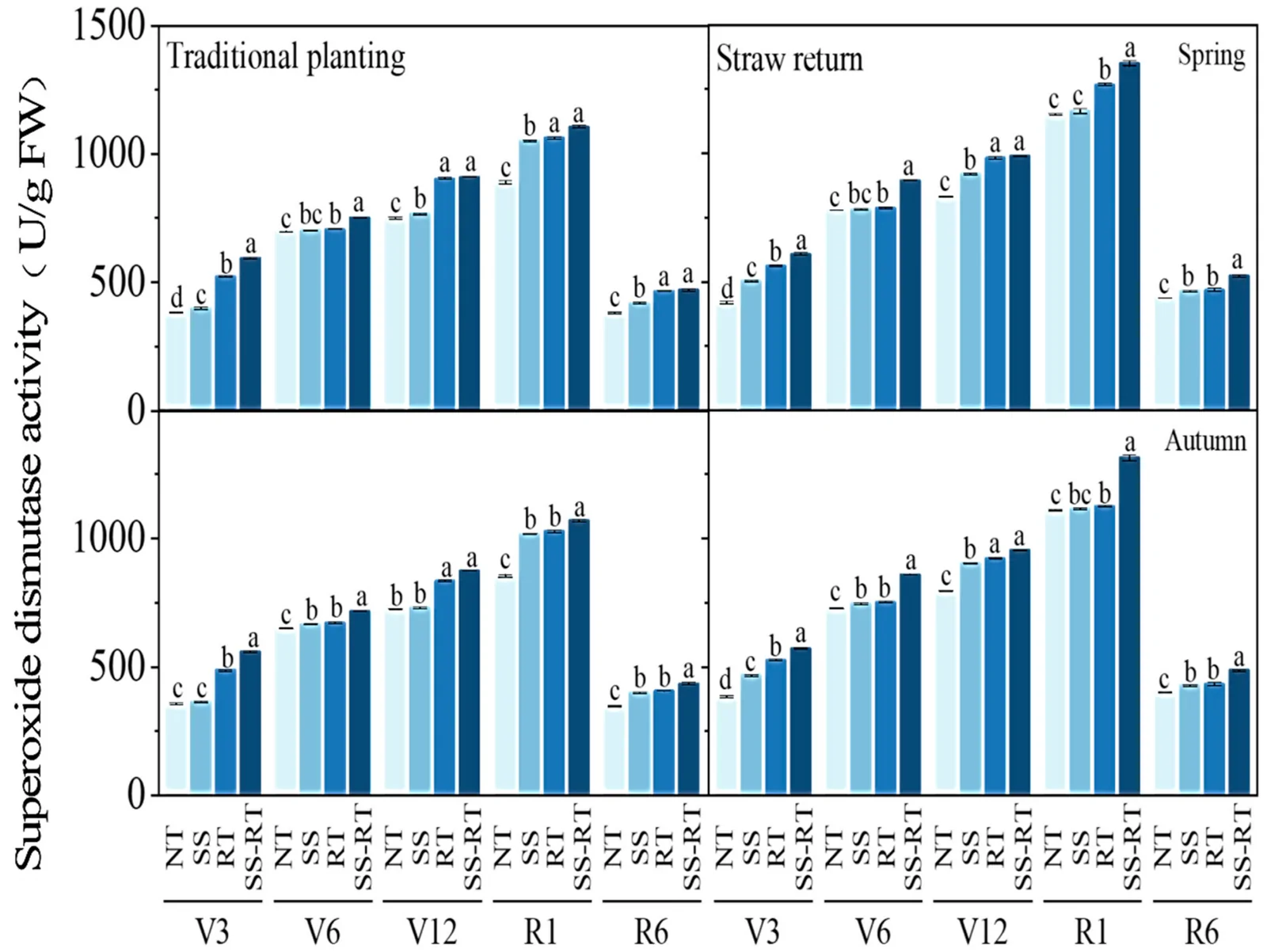
Effects of straw treatment combined with tillage methods on the superoxide dismutase activity of maize. The bars are the standard error (SE). Tillage practice was assigned to sub-plots with four levels: NT: no-tillage (0 cm), RT: rotary tillage (25 cm), SS: sub-soiling (35 cm), and SS-RT: sub-soiling followed by rotary tillage (35 cm + 25 cm). Different lower-case letters for the same growth stage between different tillage methods indicate significant differences at *p* < 0.05 (*n* = 3).

Over the spring growing period, leaf superoxide dismutase (SOD) activity rose and then declined, peaking at the R1 stage (Figure 10). At V6, R1, and R6, SOD activity under SS-RT was significantly higher than under RT, SS, and NT within TP (*p* < 0.05), whereas RT and SS did not differ (*p* > 0.05). At V3 and V12, the overall pattern SS-RT > RT > SS > NT was again significant (*p* < 0.05). During the autumn season, mean leaf SOD activity was 697.59 U g^−1^ under TP and 794.49 U g^−1^ under SR, an increase of 13.89% with SR (*p* < 0.05). Mean activities at V3, V6, V12, R1, and R6 were 499.18, 762.88, 883.02, 1131.22, and 453.90 U g^−1^, respectively, while seasonal means for SS-RT, RT, SS, and NT were 820.28, 773.64, 717.17, and 673.07 U g^−1^. Relative to TP, SR increased SOD activity by 9.90% (SS-RT), 0.37% (RT), 9.61% (SS), and 12.65% (NT). Across the seasons, SR consistently produced higher plant SOD activity than TP (*p* < 0.05). Leaf SOD activity in autumn exceeded that in spring by 5.65%, and, relative to NT, SS, RT, and SS-RT, raised SOD activity by 7.50%, 14.53%, and 22.67%, respectively.

**Figure 11:**
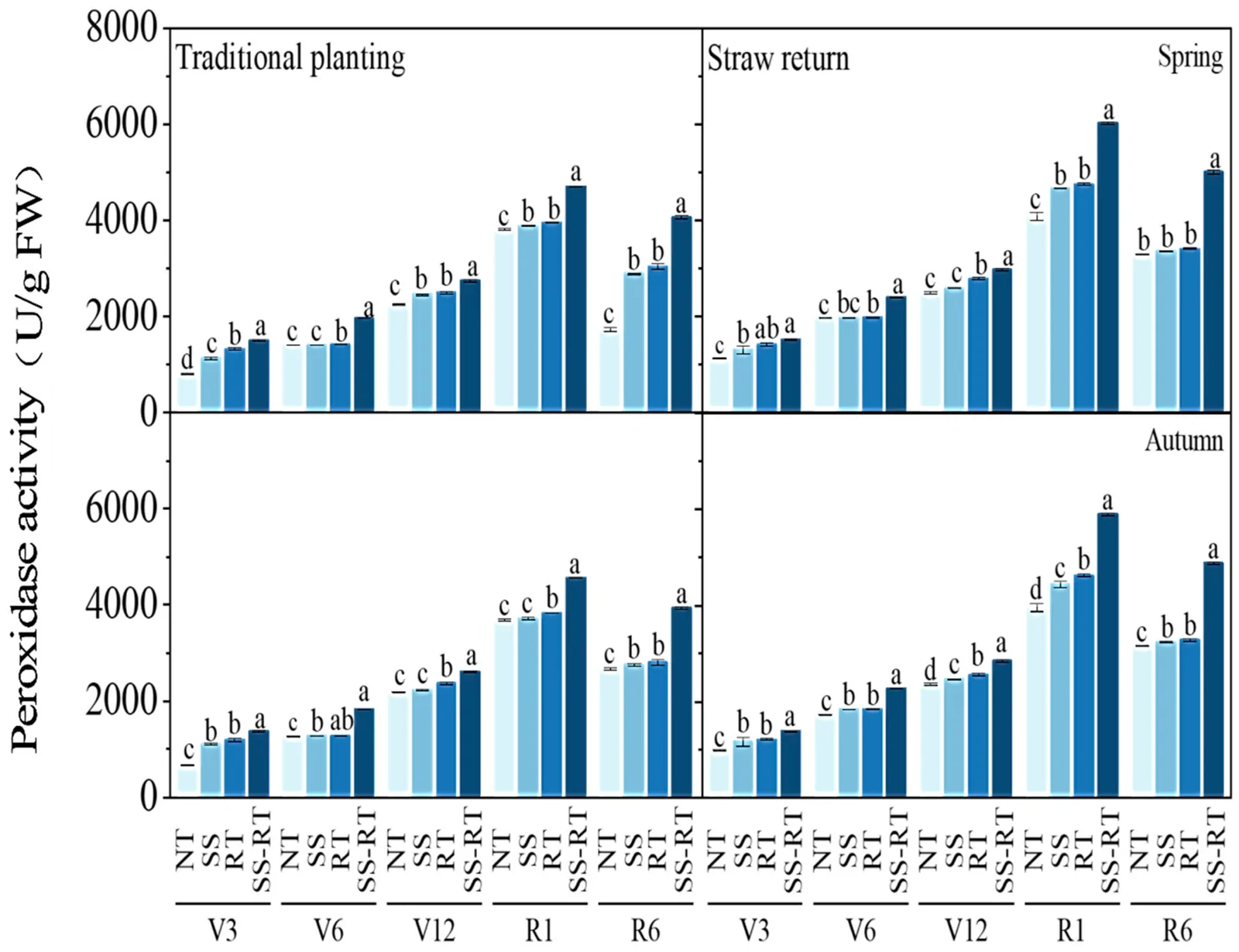
Effects of straw treatment combined with tillage methods on the peroxidase activity of maize. The bars are the standard error (SE). Tillage practice was assigned to sub-plots with four levels: NT: no-tillage (0 cm), RT: rotary tillage (25 cm), SS: sub-soiling (35 cm), and SS-RT: sub-soiling followed by rotary tillage (35 cm + 25 cm). Different lower-case letters for the same growth stage between different tillage methods indicate significant differences at *p* < 0.05 (*n* = 3).

Leaf peroxidase (POD) activity was significantly influenced by the interaction between SR and tillage practice (*p* < 0.05). In spring maize, POD activity under SR versus TP was 1192.50 vs. 1087.75 U g^−1^ at V3, 1925.33 vs. 1411.33 U g^−1^ at V6, 2558.75 vs. 2347.75 U g^−1^ at V12, 4726.42 vs. 3948.92 U g^−1^ at R1, and 3637.83 vs. 3041.75 U g^−1^ at R6; thus, SR exceeded TP at every stage. Relative to TP, SR increased POD activity by 17.09% (SS-RT), 15.20% (RT), 15.69% (SS), and 14.27% (NT); within each tillage system, SR was always significantly higher than TP (*p* < 0.05). During autumn, same pattern was observed: mean POD activity under SR and TP was 2958.18 and 2449.65 U g^−1^, respectively, representing a 17.19% increase with SR. Compared with TP, SR raised POD activity by 11.23% (V3), 25.84% (V6), 8.35% (V12), 16.17% (R1), and 22.22% (R6). The two-season results showed that straw return, tillage practice, and season each exerted a significant effect on leaf POD activity (*p* < 0.05) (Figure 11). Within each tillage practice, straw return increased POD activity relative to traditional planting to remove the straw by 20.15% for SS-RT, 15.20% for RT, 15.69% for SS, and 14.27% for NT.

**Figure 12:**
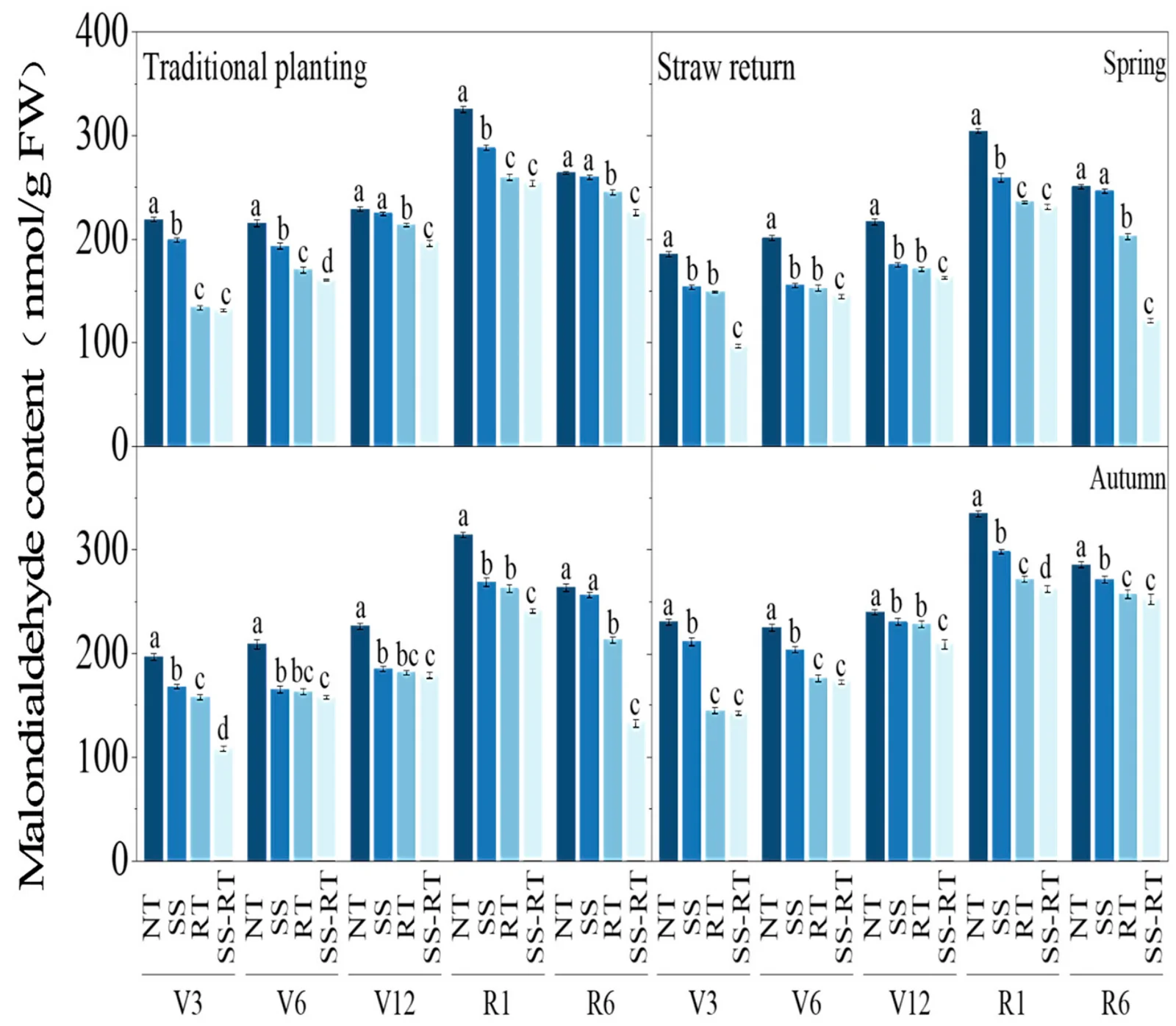
Effects of straw treatment combined with tillage methods on the malondialdehyde content of maize. The bars are the standard error (SE). Tillage practice was assigned to sub-plots with four levels: NT: no-tillage (0 cm), RT: rotary tillage (25 cm), SS: sub-soiling (35 cm), and SS-RT: sub-soiling followed by rotary tillage (35 cm + 25 cm). Different lower-case letters for the same growth stage between different tillage methods indicate significant differences at *p* < 0.05 (*n* = 3).

During the spring maize season, leaf malondialdehyde (MDA) content averaged 190.78 nmol g^−1^ under SR and 220.36 nmol g^−1^ under TP, representing a significant 13.42% reduction with SR (*p* < 0.05). Relative to NT, MDA under TP decreased by 22.39% with SS-RT, 18.32% with RT, and 6.94% with SS; the corresponding reductions under SR were 34.67%, 21.32%, and 14.61%. At every developmental stage and within each tillage practice, MDA content was significantly lower under SR than TP (*p* < 0.05), with reductions of 14.27% (V3), 11.63% (V6), 15.93% (V12), 8.59% (R1), and 17.47% (R6). In autumn maize, leaf MDA concentrations averaged 202.61 nmol g^−1^ under SR and 232.43 nmol g^−1^ under TP. Mean values at V3, V6, V12, R1, and R6 were 170.12, 184.12, 209.94, 281.85, and 241.57 nmol g^−1^, respectively. Throughout the season, SS-RT, RT, and SS consistently showed lower MDA than NT. Within each tillage system, MDA was always lower under SR than TP (*p* < 0.05); within each straw regime, SS-RT produced significantly lower MDA than RT, SS, and NT (*p* < 0.05). Across the two seasons (Figure 12), SR reduced MDA by an average of 13.12% compared with TP (*p* < 0.05). Seasonally, spring maize exhibited 5.49% less MDA than autumn maize.

**Figure 13:**
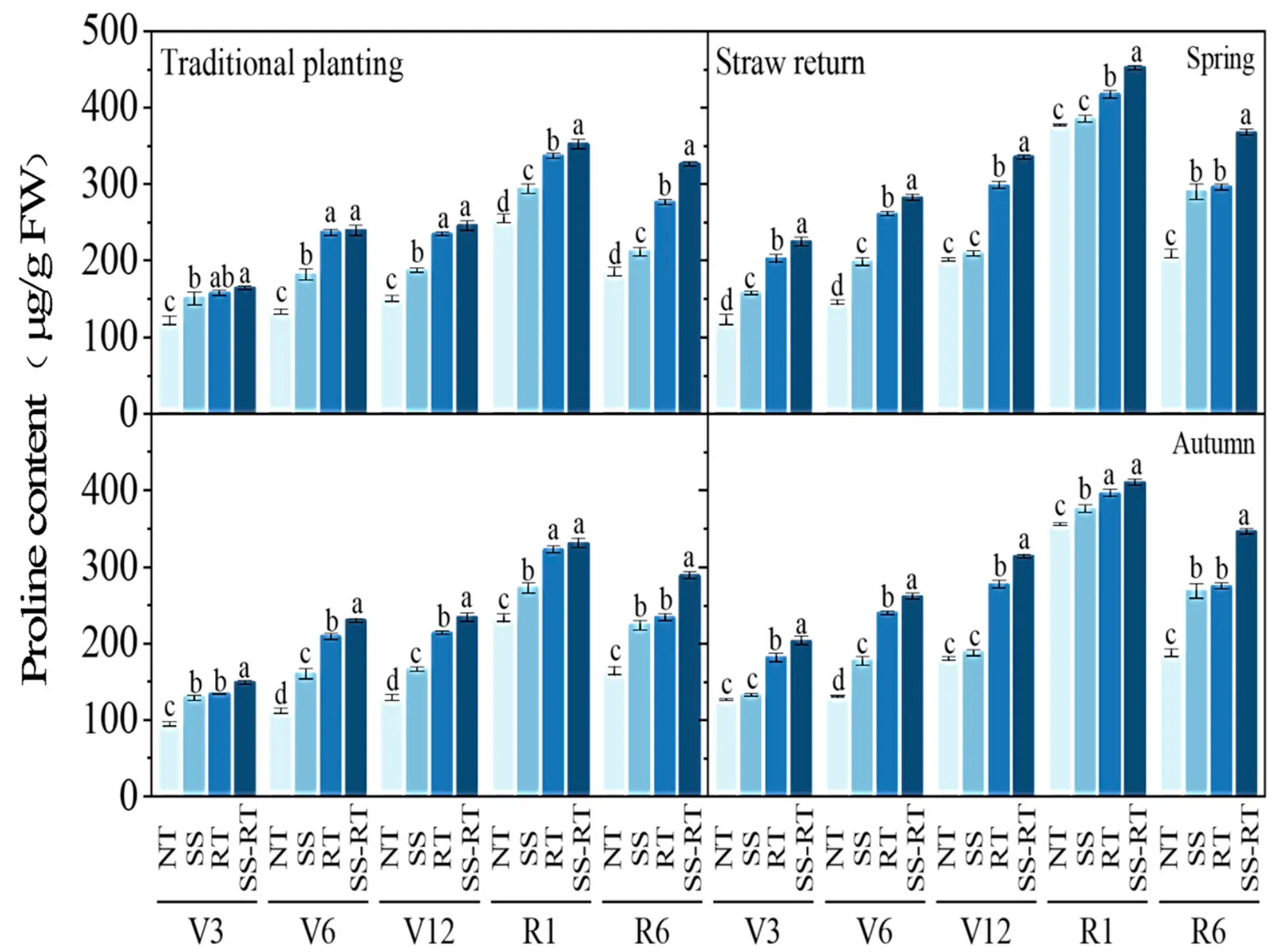
Effects of straw treatment combined with tillage methods on the proline content of maize. The bars are the standard error (SE). Tillage practice was assigned to sub-plots with four levels: NT: no-tillage (0 cm), RT: rotary tillage (25 cm), SS: sub-soiling (35 cm), and SS-RT: sub-soiling followed by rotary tillage (35 cm + 25 cm). Different lower-case letters for the same growth stage between different tillage methods indicate significant differences at *p* < 0.05 (*n* = 3).

During the spring growing period, leaf proline (Pro) content increased to a maximum at the R1 stage and declined thereafter (Figure 13). Under traditional planting to remove the straw, Pro concentrations at V3 and V6 were significantly higher in the SS-RT and RT treatments than in SS and NT (*p* < 0.05), while SS and NT did not differ (*p* > 0.05); at V12, R1 and R6, SS-RT and RT were statistically indistinguishable (*p* > 0.05). Under straw return, Pro content at V3, V6, V12, and R1 was significantly greater in SS-RT than in RT, SS, and NT. In autumn maize, mean leaf Pro concentrations at V3, V6, V12, R1, and R6 were 163.16, 210.16, 233.07, 358.92, and 270.61 μg g^−1^, respectively. Averaged across stages, SR increased Pro to 272.00 μg g^−1^, 18.25% higher than the 222.37 μg g^−1^ observed under TP. Throughout the autumn season, SR raised Pro relative to TP by 16.09% (V3), 10.82% (V6), 21.56% (V12), 24.13% (R1), and 14.01% (R6). Across the two seasons (Figure 13), SR enhanced Pro by 22.83% (V3), 12.89% (V6), 28.17% (V12), 32.25% (R1), and 17.30% (R6) within the same tillage practice (*p* < 0.05). These results demonstrate that Pro accumulation is significantly modulated by the interactions among straw management, tillage practice, and growing season.

### 3.8. Maize Production

In spring-grown maize, SS-RT consistently produced a significantly greater kernel number per ear (KNE), thousand-grain weight (TGW), and grain yield than RT, SS, and NT under both TP and SR. Compared with NT, SS-RT increased the KNE under TP; corresponding TGW gains were TP and SR, while yield was enhanced under TP and SR treatments. For autumn maize, SR, tillage, and their interaction all had significant effects on KNE, TGW, and yield (*p* < 0.05). Within each straw-management regime, the ranking SS-RT > RT > SS > NT was maintained. Under TP, SS-RT surpassed RT; SS and NT significantly improved the KNE, TGW, and grain yield. Across the seasons, SR increased KNE, TGW, and grain yield relative to TP. Straw return and tillage each affected KNE, while their main and interactive effects significantly influenced TGW and yield (*p* < 0.05); spring maize achieved higher KNE, TGW, and yield than autumn maize (Table 5).

### 3.9. Correlation Analysis

**Figure 14:**
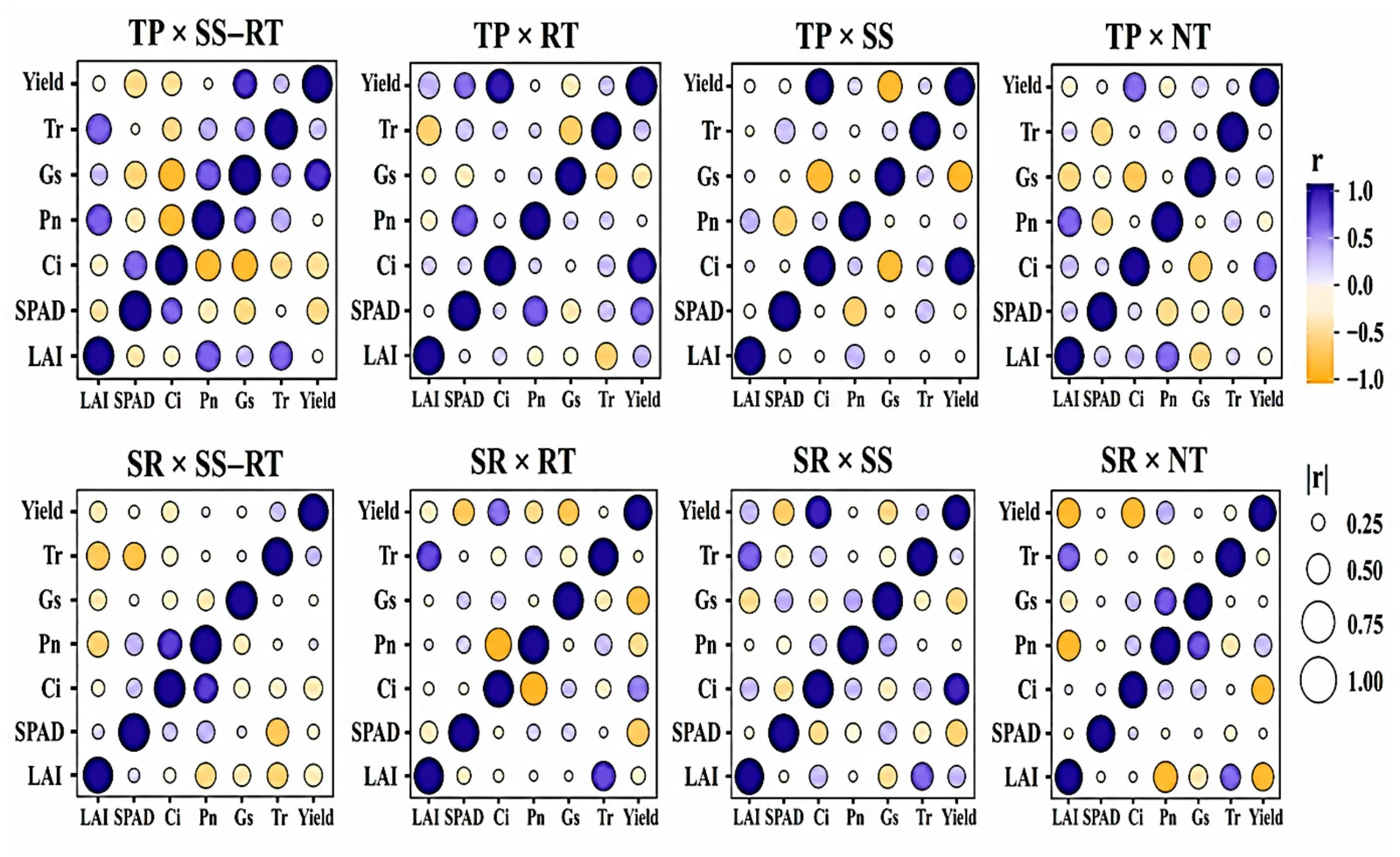
Correlation analysis of yield with photosynthetic physiological characteristics.

Across the seasons, the LAI exhibited significant (*p* < 0.05) positive correlations with Ci, Pn, Gs, Tr, plant height, stem diameter, ear height, ear length, ear diameter, and grain yield (Figure 14). SPAD showed an almost identical pattern of positive associations, yet its correlation with the LAI itself was weak (|r| < 0.2). The gas-exchange variables Ci, Pn, Gs, and Tr were strongly inter-correlated, and each was positively linked to yield and to all five morphological traits listed above (*p* < 0.05). Ear-tip length displayed the opposite trend, being negatively correlated with SPAD, the four gas-exchange traits, and yield, as well as with plant height, stem diameter, ear height, ear length, and ear diameter (*p* < 0.05). Finally, every morphological trait measured was positively associated with yield, whereas the relationship between plant height and ear diameter was comparatively weak. Together, these correlations indicate that a larger, greener canopy and enhanced gas exchange promote ear development and final yield, while longer ear tips (indicative of poor pollination or kernel set) are detrimental.

## 4. Discussion

### 4.1. Optimization of Soil Physical Environment

This study demonstrates that the combination of straw return and deep tillage (SS + RT) creates a more favorable edaphic environment than single tillage practices. Although traditional rotary tillage (RT) effectively incorporates straw into the shallow layer (0–15 cm), it tends to form a distinct plough pan that physically restricts root penetration into deeper soil profiles [20,29]. In contrast, the SS-RT treatment, which fractures the soil to a depth of 35 cm, likely disrupts this compacted layer. Moreover, the interaction between straw return and tillage intensity plays a critical role in regulating soil water dynamic. In contrast, sub-soiling alleviates sub-soil compaction by disrupting dense soil layers, thereby improving soil aeration, water infiltration, root penetration, and access to deeper soil moisture [19,20,21,22]. Integrating deep sub-soiling with rotary tillage combines the advantages of both practices by simultaneously alleviating soil compaction and creating a well-prepared seedbed, providing favorable conditions for crop establishment and growth. No-tillage (NT) provides notable soil conservation benefits, in the clay soils of the present study region, potentially due to increased mechanical impedance and lower soil temperatures relative to tilled plots. Conversely, deep straw incorporation under SS-RT likely enhances straw-soil contact and accelerates decomposition compared with surface mulching, resulting in greater carbon and nitrogen release in sub-soil layers [5,7]. These findings are consistent with Wang et al. [11], who reported that deep straw return significantly increases soil porosity and water storage capacity in the 20–40 cm layer, thereby providing a critical buffer against water stress during the grain-filling period.

### 4.2. Regulation of Photosynthetic Capacity and Delayed Senescence

Maintaining a high LAI and Pn during the reproductive stage is critical for achieving high maize yield. The present study demonstrated that the SS-RT treatment significantly delayed leaf senescence, as evidenced by higher SPAD values and net photosynthetic rates from silking (R1) to maturity (R6). This pronounced “stay-green” effect is likely attributed to the enhanced root system described above, which ensures a sustained supply of water and nutrients, particularly nitrogen, to the canopy during late growth stages [3]. Chen et al. [7] found that increasing topsoil depth significantly enhanced photosynthesis and nitrate content, which in turn up regulated the activity of photosynthetic enzymes such as Rubisco and PEPCase in the leaves. In this study, the higher G_s_ and C_i_ under SS-RT treatment suggest that stomatal factors played a key role in maintaining high photosynthesis. Unlike NT, where sub-soil compaction might induce water stress and stomatal closure, the deep-tilled profile of SS-RT maintained better soil water content, allowing stomata to remain open for CO_2_ intake without excessive dehydration [14]. Thus, the synergy of sub-soiling and straw return effectively mitigates the “source” limitation during grain filling.

### 4.3. Enhancement of Antioxidant Defense Metabolism

Reactive oxygen species (ROS) metabolism plays a central role in regulating plant senescence and stress response. In the present study, malondialdehyde (MDA) accumulation, a key indicator of membrane lipid peroxidation, was significantly lower under the SS-RT treatment, while the activities of major antioxidant enzymes (SOD, POD, CAT) were markedly enhanced. This suggests that the combined tillage practice primed the antioxidant defense system of maize. The enhancement of antioxidant capacity under SS-RT likely arises from two complementary mechanisms. First, sub-soiling alleviates soil physical constrains, such as sub-soil compaction and hypoxia, thereby reducing basal ROS production in leaves [14]. Second, straw decomposition releases humic substances and signaling molecules that can stimulate the synthesis and activity of antioxidant enzymes. Consistent with this, Xu et al. [3] reported that optimizing the soil environment through organic amendments can balance endogenous hormones (e.g., increasing cytokinins, decreasing ABA), which directly regulate the expression of defense-related enzymes. By maintaining cellular membrane integrity, the SS-RT treatment protected the photosynthetic apparatus from oxidative damage, ensuring that the “physiological machinery” remained functional longer into the grain-filling period [11,30,31]. Several studies have shown that integrating straw return with appropriate tillage practices enhances these physiological processes. For example, ridge–furrow straw management significantly increased SOD and CAT activities while reducing malondialdehyde (MDA) accumulation and ROS levels, thereby protecting the photosynthetic apparatus in semi-arid maize systems [23]. Similarly, long-term straw return combined with an appropriate nitrogen application increased SPAD values by approximately 2%, net photosynthetic rate (Pn) by about 8%, dry matter accumulation, and grain yield by nearly 15% compared with conventional management [24].

### 4.4. Synergistic Yield Formation

Grain yield ultimately represents the most integrative indicator of agronomic performance. In the present study, the SS-RT treatment with straw return achieved the highest yield, primarily due to the significant increases in 1000-grain weight and kernel number per ear. These outcomes are consistent with the source–sink theory: the “source” (leaves) remained active longer due to delayed senescence, and the “flow” (transport) was supported by a healthy vascular system in the stalks. Feng et al. [19] reported that sub-soiling improves stalk vascular transport efficiency, which facilitates the translocation of photo assimilates to the ear. Complete residue return drawbacks include temporary N immobilization, sowing and residue-management difficulties, phytotoxicity, disease risks, and possible increases in N_2_O emissions; however, these effects depend strongly on residue quality, C ratio, soil conditions, and management practices [11]. Moreover, our results contradict that straw return might decrease yield due to nitrogen immobilization. This discrepancy is likely attributed to the use of the “Entek” slow-release fertilizer, which synchronized nitrogen supply with crop demand, coupled with the deep tillage that minimized nutrients stratifications within the soil profile [11]. Conventional rotary tillage effectively prepares the seedbed and incorporates surface residues but, when practiced continuously, may lead to the formation of a compact plough layer that restricts root growth and water movement [20]. In contrast, sub-soiling alleviates sub-soil compaction by disrupting dense soil layers, thereby improving soil aeration, water infiltration, root penetration, and access to deeper soil moisture [19,21,22]. Integrating deep sub-soiling with rotary tillage combines the advantages of both practices by simultaneously alleviating soil compaction and creating a well-prepared seedbed, providing favorable conditions for crop establishment and growth. Furthermore, Zhang et al. [26] demonstrated that four years of straw return increased soil urease, phosphatase, and invertase activities by up to 44.3%, thereby improving nutrient cycling and supporting higher photosynthetic capacity and maize productivity. Collectively, these results indicate that the SS-RT system represents an integrated strategy that simultaneously improves soil structure, sustains physiological activity, and maximizes grain yield in subtropical maize production systems.

## 5. Conclusions

Integrating straw return with sub-soiling plus rotary tillage (SS-RT) is an effective management strategy for intensive maize production in subtropical double-cropping systems. This integrated practice enhances photosynthetic performance, delays leaf senescence, strengthens antioxidant defense mechanisms, and promotes greater dry matter accumulation, resulting in superior grain yield and yield components. Compared with conventional residue removal and no-tillage, the combined approach provides a sustainable solution for improving maize productivity while facilitating environmentally friendly crop residue management. These findings support the adoption of straw return coupled with deep–shallow tillage as a practical strategy for improving the photosynthetic capacity, crop performance, antioxidant defense system, and maize productivity while providing an effective alternative to open-field straw burning.

## Figures and Tables

**Table 1:** Effect of straw treatment combined with tillage treatments on the intercellular carbon dioxide concentration of maize.

Treatment		Spring	Autumn	Mean
Traditional planting	NT	103.79 ± 0.40 d	102.94 ± 0.25 d	103.37 ± 0.32 d
	SS	129.07 ± 0.26 c	119.00 ± 0.39 c	124.04 ± 0.27 c
	RT	131.77 ± 0.27 b	123.54 ± 0.52 b	127.66 ± 0.31 b
	SS-RT	138.71 ± 0.77 a	130.05 ± 0.44 a	134.38 ± 0.60 a
Straw return	NT	124.49 ± 0.44 d	120.76 ± 0.30 d	122.63 ± 0.36 d
	SS	152.68 ± 0.34 c	141.46 ± 0.02 c	147.07 ± 0.16 c
	RT	161.49 ± 0.46 b	144.58 ± 0.08 b	153.04 ± 0.19 b
	SS-RT	165.57 ± 0.36 a	152.80 ± 0.23 a	159.18 ± 0.11 a
*p* values				
ST		0.0001	0.0002	0.0001
TM		0.0001	0.0001	0.0001
ST × TM		0.0001	0.0001	0.0001

Mean ± SE; different lower-case letters within a column indicate significant differences at the 5% probability level with the least significant difference (LSD) test. ST: straw return treatment, TM: traditional planting method to remove the straw. ST and TM analyses were performed separately.

**Table 2:** Effect of straw treatment combined with tillage treatments on the net photosynthetic rate of maize.

Treatment		Spring	Autumn	Mean
Traditional planting	NT	33.07 ± 0.13 d	32.35 ± 0.22 d	32.71 ± 0.05 d
	SS	35.74 ± 0.11 c	34.69 ± 0.15 c	35.22 ± 0.14 c
	RT	36.63 ± 0.13 b	35.79 ± 0.20 b	36.21 ± 0.09 b
	SS-RT	39.22 ± 0.50 a	39.08 ± 0.20 a	39.15 ± 0.06 a
Straw return	NT	35.95 ± 0.47 d	35.43 ± 0.12 d	35.69 ± 0.09 d
	SS	38.15 ± 0.29 c	38.01 ± 0.13 c	38.08 ± 0.01 c
	RT	39.73 ± 0.32 b	38.73 ± 0.07 b	39.23 ± 0.07 b
	SS-RT	42.07 ± 0.43 a	41.77 ± 0.22 a	41.92 ± 0.05 a
*p* values				
ST		0.0018	0.0001	0.0003
TM		0.0001	0.0001	0.0001
ST × TM		0.7798	0.4104	0.9461

Mean ± SE; different lower-case letters within a column indicate significant differences at the 5% probability level with the least significant difference (LSD) test. ST: straw return treatment, TM: traditional planting method to remove the straw. ST and TM analyses were performed separately.

**Table 3:** Effect of straw treatment combined with tillage treatments on the stomatal conductance of maize.

Treatment		Spring	Autumn	Mean
Traditional planting	NT	0.22 ± 0.003 d	0.20 ± 0.001 d	0.21 ± 0.002 d
	SS	0.24 ± 0.004 c	0.23 ± 0.002 c	0.23 ± 0.003 c
	RT	0.25 ± 0.003 b	0.24 ± 0.003 b	0.24 ± 0.003 b
	SS-RT	0.28 ± 0.005 a	0.26 ± 0.004 a	0.27 ± 0.004 a
Straw return	NT	0.27 ± 0.002 d	0.26 ± 0.004 c	0.26 ± 0.003 c
	SS	0.31 ± 0.002 c	0.29 ± 0.001 b	0.30 ± 0.001 b
	RT	0.32 ± 0.002 b	0.30 ± 0.003 b	0.31 ± 0.002 b
	SS-RT	0.34 ± 0.001 a	0.33 ± 0.003 a	0.34 ± 0.002 a
*p* values				
ST		0.0030	0.0037	0.0022
TM		0.0001	0.0001	0.0001
ST × TM		0.0261	0.2426	0.0294

Mean ± SE; different lower-case letters within a column indicate significant differences at the 5% probability level with the least significant difference (LSD) test. ST: straw return treatment, TM: traditional planting method to remove the straw. ST and TM analyses were performed separately.

**Table 4:** Effect of straw treatment combined with tillage treatments on the transpiration rate of maize.

Treatment		Spring	Autumn	Mean
Traditional planting	NT	2.39 ± 0.006 d	2.18 ± 0.019 d	2.28 ± 0.011 d
	SS	2.60 ± 0.020 c	2.27 ± 0.006 c	2.44 ± 0.012 c
	RT	2.71 ± 0.012 b	2.42 ± 0.010 b	2.57 ± 0.011 b
	SS-RT	3.01 ± 0.025 a	2.81 ± 0.010 a	2.91 ± 0.011 a
Straw return	NT	2.84 ± 0.012 d	2.70 ± 0.011 c	2.77 ± 0.010 c
	SS	3.10 ± 0.015 c	2.84 ± 0.035 b	2.97 ± 0.010 b
	RT	3.22 ± 0.017 b	3.01 ± 0.014 b	3.11 ± 0.015 b
	SS-RT	3.57 ± 0.017 a	3.28 ± 0.017 a	3.43 ± 0.017 a
*p* values				
ST		0.0004	0.0004	0.0001
TM		0.0001	0.0001	0.0001
ST × TM		0.0183	0.0391	0.1768

Mean ± SE, different lower-case letters within a column indicate significant differences at the 5% probability level with the least significant difference (LSD) test. ST: straw return treatment, TM: traditional planting method to remove the straw. ST and TM analyses were performed separately.

**Table 5:** Effects of straw treatment combined with tillage methods on maize yield and yield components.

Treatment		KNE (Grain/Ear)		TGW (g)			Yield (kg/ha)			
		Spring	Autumn	Mean	Spring	Autumn	Mean	Spring	Autumn	Mean
Traditional planting	SS-RT	511 ± 2.65 a	499 ± 3.01 a	505 ± 3.50 a	338.7 ± 2.30 a	298.1 ± 1.81 a	318.4 ± 2.31 a	9082 ± 23.01 a	8463 ± 32.63 a	8773 ± 29.71 a
	RT	470 ± 3.60 b	480 ± 2.51 b	475 ± 3.60 b	324.9 ± 2.22 b	288.3 ± 1.40 b	306.6 ± 2.66 b	8749 ± 30.01 b	8095 ± 31.22 b	8422 ± 27.31 b
	SS	429 ± 2.01 c	449 ± 2.63 c	439 ± 2.36 c	309.5 ± 2.43 c	270.6 ± 1.31 c	290.1 ± 1.54 c	8265 ± 22.61 c	7458 ± 29.24 c	7862 ± 25.67 c
	NT	388 ± 2.91 d	441 ± 2.66 d	415 ± 2.44 d	279.9 ± 1.55 d	259.8 ± 1.26 c	269.9 ± 1.35 d	7781 ± 19.96 d	7122 ± 27.36 d	7452 ± 24.44 d
Straw return	SS-RT	520 ± 3.71 a	520 ± 3.31 a	520 ± 3.04 a	338.4 ± 2.44 a	314.8 ± 2.08 a	326.6 ± 2.38 a	9493 ± 24.63 a	8554 ± 25.39 a	9024 ± 23.48 a
	RT	490 ± 2.64 b	488 ± 2.74 b	489 ± 2.57 b	339.7 ± 2.62 a	312.4 ± 2.13 a b	326.1 ± 2.46 a	9212 ± 27.51 b	8417 ± 21.44 b	8815 ± 22.54 b
	SS	480 ± 2.11 c	471 ± 2.62 c	476 ± 2.67 c	319.5 ± 2.71 b	303.1 ± 2.33 b c	311.3 ± 3.09 b	8370 ± 29.11 c	7701 ± 22.54 c	8036 ± 25.39 c
	NT	409 ± 2.81 d	462 ± 2.61 d	438 ± 1.93 d	311.4 ± 1.01 c	289.9 ± 1.87 c	300.7 ± 3.21 c	7941 ± 21.22 d	7489 ± 23.09 d	7715 ± 24.19 d
*p* value										
ST		0.0030	0.0123	0.0204	0.0131	0.0018	0.0031	0.0015	0.0012	0.0016
TM		0.0004	0.0002	0.0003	0.0003	0.0012	0.0012	0.0028	0.0015	0.0051
ST × TM		0.0007	0.2417	0.0006	0.0005	0.0041	0.0024	0.0012	0.0012	0.0022

Note: Mean ± SE; different lower-case letters within a column indicate significant differences at the 5% probability level with the least significant difference (LSD) test. ST: straw return treatment, TM: traditional planting method to remove the straw. ST and TM analyses were performed separately.

## Data Availability

The original contributions presented in this study are included in the article. Further inquiries can be directed to the corresponding author.
